# Biosensors and Sensing Systems for Rapid Analysis of Phenolic Compounds from Plants: A Comprehensive Review

**DOI:** 10.3390/bios10090105

**Published:** 2020-08-24

**Authors:** Cristina Forzato, Veronica Vida, Federico Berti

**Affiliations:** Dipartimento di Scienze Chimiche e Farmaceutiche, Università degli Studi di Trieste, via Giorgieri 1, 34127 Trieste, Italy; cforzato@units.it (C.F.); veronica.vida@phd.units.it (V.V.)

**Keywords:** electrochemical sensors, optical sensors, phenols, sensing systems, analytical methodology, biorecognition, artificial receptors

## Abstract

Phenolic compounds are secondary metabolites frequently found in plants that exhibit many different effects on human health. Because of the relevant bioactivity, their identification and quantification in agro-food matrices as well as in biological samples are a fundamental issue in the field of quality control of food and food supplements, and clinical analysis. In this review, a critical selection of sensors and biosensors for rapid and selective detection of phenolic compounds is discussed. Sensors based on electrochemistry, photoelectrochemistry, fluorescence, and colorimetry are discussed including devices with or without specific recognition elements, such as biomolecules, enzymes and molecularly imprinted materials. Systems that have been tested on real matrices are prevalently considered but also techniques that show potential development in the field.

## 1. Introduction

The plant kingdom is one of the richest reservoirs of organic compounds of different structures, called secondary metabolites, which are quite always unique for each species.

Among the thousands of secondary metabolites present in plants, phenolic compounds are one of the wider classes synthetized. To this class of compounds belong all the ones that possess one phenol ring, such as phenolic acids and phenolic alcohols, or more than one phenol ring, which are named polyphenols. Their definition is not always so clear, since several classifications have been proposed, but according to most of the literature, they are divided into phenolic acids, lignans, stilbenes, and flavonoids [1,2,3,4]. Flavonoids can be further divided into six subclasses depending on the type of heterocycle involved: flavonols, flavones, isoflavones, flavanones, anthocyanidins, and flavanols (catechins and proanthocyanidins) [5].

Phenolic compounds are present in fruits, vegetables, nuts, seeds, flowers, and tree barks, and originate from the acetate pathway and the shikimate pathway, or the two combined. They are considered excellent antioxidants and are related to the diminishment of risks of important chronic diseases, as demonstrated by the many reviews present in the literature on their biological activity. For this reason, the food sector is developing functional foods with health benefits [6,7,8,9,10]. Moreover, in 2012, a health claim was approved (Commission Regulation EU 432/2012) on the beneficial effects of polyphenols in olive oil, thus leading to the necessity to develop rapid systems for identifying and quantifying these compounds in foods.

### 1.1. Flavonoids

**Figure 1:**
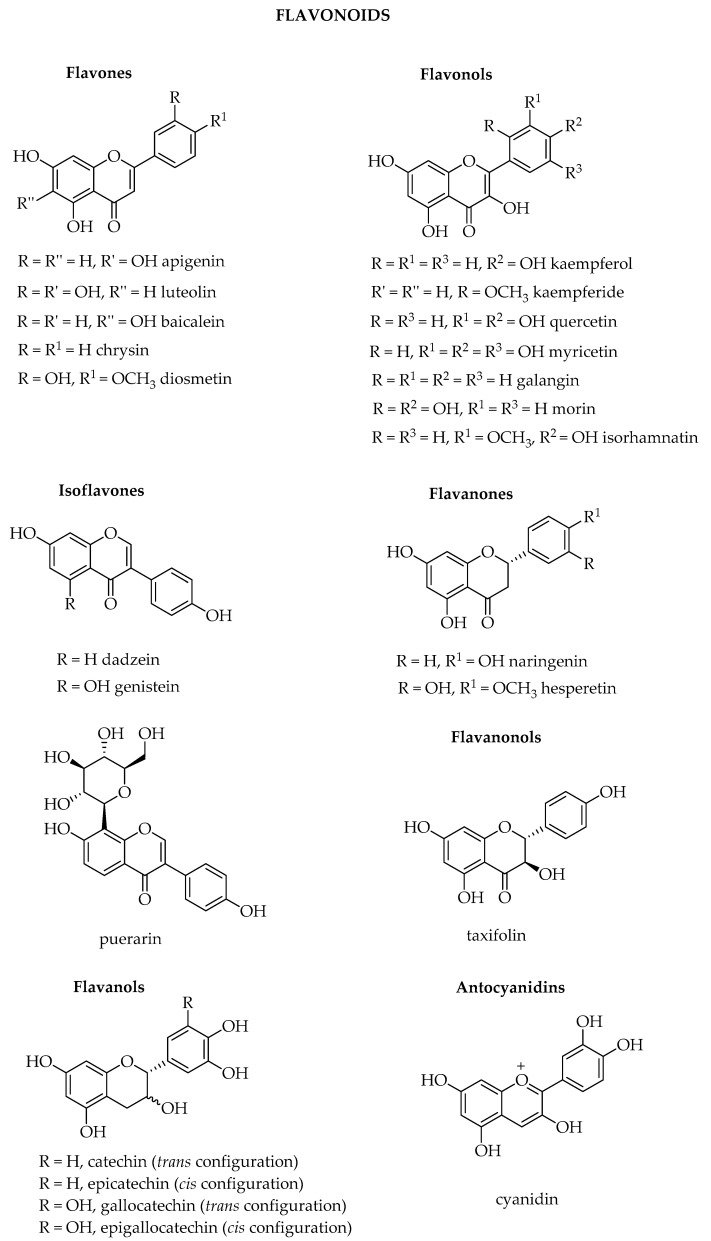
Flavonoids.

Flavonoids are the most representative class, since more than 5000 compounds have been characterized from various plants, usually serving as pigments for flower coloration to attract pollinator animals. They may also act as UV filters, chemical messengers, physiological regulators, and cell cycle inhibitors. Flavonoids exert several biological activities such as antioxidative, free radical scavenging capacity, cardioprotective, antidiabetic, anti-inflammatory, anti-allergic. More recently, flavonoids have proven to act also as anti-cancer agents [11,12]. Apigenin, luteolin, and baicalein (Figure 1) are part of the flavones subgroup of flavonoids, and are present in both vegetables and fruits, as well as in medicinal herbs. Apigenin is abundant in celery, onion, garlic, bell pepper, guava, passionflower and bilimbi fruit, while luteolin is found in high amounts in thyme, peppermint, parsley, celery, green pepper, perilla leaves, and chamomile tea. Baicalein, the aglycone of baicalin, was originally isolated from the roots of *Scutellaria baicalensis* and *Scutellaria lateriflora* but it is also reported in *Oroxylum indicum* (Indian trumpet flower) and thyme. Among the several biological activities already known, more recently it has shown anti-cancer functions with low toxicity, including proliferation inhibition, apoptosis induction, autophagy cell death, and anti-metastasis activity [13]. The isoflavones daidzein and genistein are mainly present in soy, both as free aglycones or as 7-*O*-glucosides, in the 0.1–0.4% range, depending on the variety. Isoflavones are defined phytoestrogens, since they exert estrogenic activity although in less extent with respect to the steroidal hormones. Foods, which are rich of phytoestrogens, could prevent cardiovascular diseases, protect from osteoporosis, reduce the risk of breast cancer, and exert antioxidant activity as all flavonoids in general. 

Kaempferol, quercetin, and myricetin are part of the flavonols subgroup of flavonoids, and quercetin in particular is frequently present in tissues of plants. Many fruits, vegetables, leaves, seeds, and grains contain these flavonols: red onions and kale are common foods containing appreciable amounts of quercetin, while kale, beans, tea, spinach, and broccoli are foods containing kaempferol. Naringenin, an example of flavanone, is present mainly in grapefruit but is found also in a variety of fruit and vegetables. (*S*)-naringenin is the predominant enantiomer in orange, apple, and tomato juices but its racemization can occur quickly [14]. As for other flavonoids, naringenin has antioxidant, antimicrobial, anticancer, antimutagenic, anti-inflammatory, cholesterol lowering, and neuroprotective effects. Naringenin can undergo several other reactions leading to the biosynthesis of catechins and epicatechins, of the flavanols subgroup, which differ for the stereochemistry of one of the two chiral stereocentres. Catechins have a *trans* configuration while epi-catechins have the *cis* configuration. Green tea is particularly rich of these compounds and of gallic acid derivatives. Galloylated catechins and flavonol 3-*O*-glycosides are characteristic astringent taste compounds in tea (*Camellia sinensis*). The mechanism involved in the formation of these metabolites remains unknown in tea plants [15]. Moreover, catechins, according to the presence of the gallic group at position 3 of the pyranose ring, can be divided into non-gallated compounds, such as (+)-catechin, (−)-epicatechin, (+)-gallocatechin, (−)-epigallocatechin, and gallated molecules such as (−)-epicatechin-3-gallate and (−)-epigallocatechin-3-gallate; gallated catechins are abundant in tea, accounting for about 70% of total catechins content [16].

(+)-Taxifolin (also known as dihydroquercetin) has two chiral centers, but is mainly present in nature as the (2*R*, 3*R*) isomer and is found in many plants. It was first isolated from Douglas fir bark (*Pseudotsuga taxifolia* (Lindl.) Britton) and later on in Dahurian and Siberian larch (*Larix sibirica* Ledeb. and *Larix gmelinii* (Rupr.) Kuzen.); it has several biological activities, such as anti-inflammatory, anticancer, antimicrobial, antioxidative and prevents cardiovascular and liver disorders [17].

### 1.2. Stilbenes and Lignans

**Figure 2:**
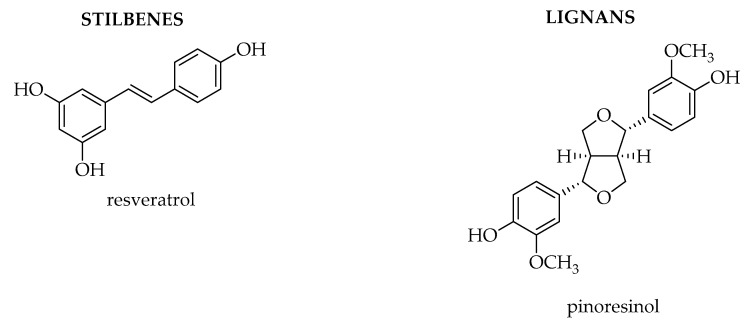
Stilbenes and lignans.

The most popular stilbene is resveratrol (Figure 2) which is found in grape and wine as well as in other fruits like blueberries, raspberries, mulberries, and peanuts. It is commercialized as a food supplement being isolated from grape skins and *Polygonum causpidatum* and it shows different health benefits, since it exerts antioxidant, anti-inflammatory, and anticancer activity and also prevents cardiovascular diseases.

Lignans derive from the hydroxycinnamic acids, which are converted into the corresponding alcohols (Figure 2). The alcohols can subsequently dimerize to give lignans or polymerize to give lignin. Several lignans are present in plants: they are widespread in vegetables, berries, and other fruits and they occur mainly as glycosides in foods. As an example, (+)-pinoresinol is a lignan present in olives, as well as in other foods: several studies have highlighted beneficial effects of (+)-pinoresinol and 1-acetoxypinoresinol which are characteristics of extra virgin olive oils.

### 1.3. Phenolic Acids 

**Figure 3:**
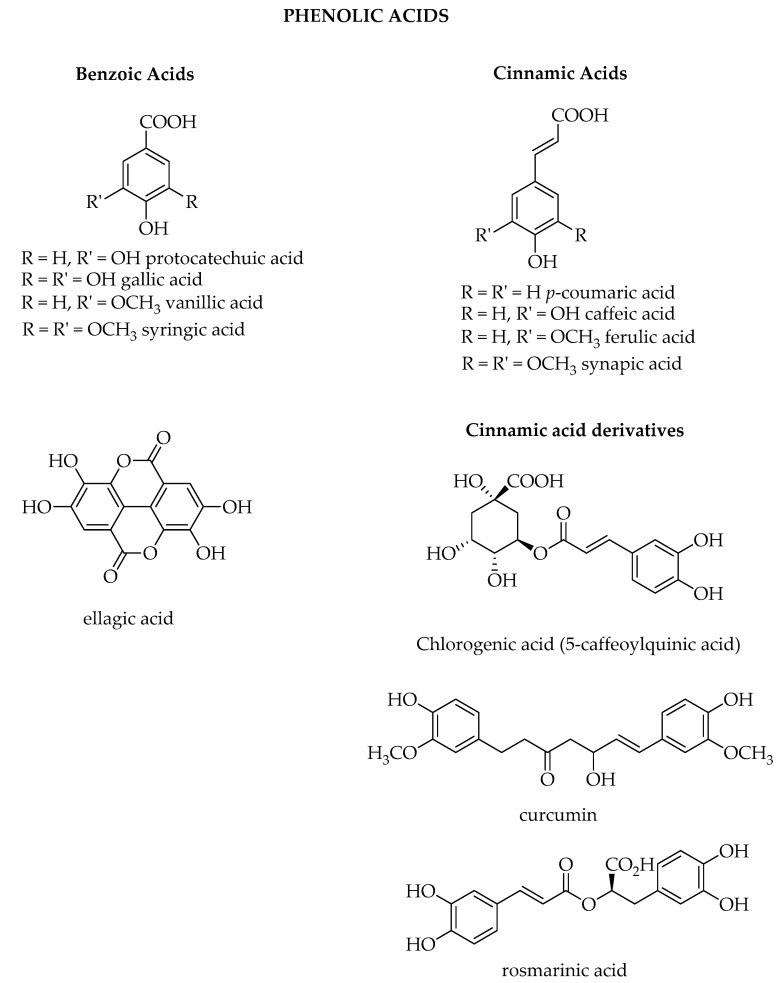
Phenolic acids.

In the class of phenolic acids, we can find benzoic acids and cinnamic acids as well as cinnamic acid derivatives such as chlorogenic acid, rosmarinic acid, and curcumin (Figure 3). Phenolic acids are described in a recent review where bioavailability and health benefits are also considered [18].

Protocatechuic acid, vanillic acid, syringic acid, and gallic acid are the most common hydroxybenzoic acids found in vegetables and several biosynthesis are present depending on the organism and sometime for the same organism different pathways can be present. In particular, gallic acid derives from the oxidation of 3-dehydroshikimic acid and it is mainly present in hydrolysable tannins [19]. Gallic acid is the starting unit for the biosynthesis of ellagic acid which is found in oak species as well as in chestnuts, walnuts, raspberries, strawberries, and grapeseed.

Phenylalanine (the essential amino acid from the shikimate pathway) is the precursor of the cinnamic acids [20], and the introduction of the double bond on the alkyl chain is due to an enzymatic reaction with the enzyme phenyl ammonia lyase. Several hydroxylation steps on the aromatic ring and subsequent methylation by S-adenosyl-methionine give origin to the different cinnamic acids *p*-coumaric acid, caffeic acid, ferulic acid, and sinapic acid. A condensation reaction of the cinnamic acids with quinic acid (derived from reduction of 3-dihydroquinic acid) gives origin to the class of chlorogenic acids, which are widely present in the plant kingdom. Different chlorogenic acids can be present depending on the hydroxyl groups of the quinic acid used for the esterification reactions and on the number of cinnamic acids used. Monoesters, diesters, and even triesters are in fact part of the chlorogenic acids family and coffee is particularly rich of these compounds which exhibit several health benefits [21,22,23]. *p*-coumaroyl-CoA and 4-hydroxyphenyl lactic acid are the precursors of rosmarinic acid, first isolated from *Rosmarinus officinalis* with antioxidant, antimicrobial, and anti-inflammatory properties [24]. Feruloyl-CoA is the starting unit for the synthesis of curcumin where a malonyl-CoA unit and a second molecule of feruloyl-CoA are involved through a Claisen reaction and a subsequent decarboxylation reaction. It is the principal component in *Curcuma longa* and it is particularly studied because of its anti-inflammatory, antiulcer, and anticancer properties [25].

### 1.4. Other Phenolic Compounds

**Figure 4:**
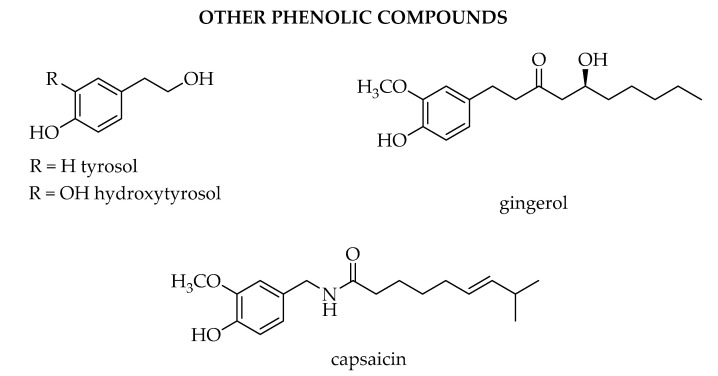
Other phenolic compounds.

Among other phenols present in plants (Figure 4), we find capsaicin, which is part of the capsaicinoid chemical compounds, responsible of the pungency and “hotness” of the *Capsicum* family of plants. It is an amide produced from vanillamine and 8-methyl-6-nonenoyl-CoA and although the plant seems to produce it for defense against certain mammals and fungi, it exhibits several positive effects on human health as antimutagenic, anticarcinogenic, anti-inflammatory, and antitumoral properties [26]. Its application field is in pharmaceutical products to relief pain as well as an active ingredient in pepper spray for self-defense.

In the gingerol biosynthesis, a thioester of hexanoic acid (hexanoyl-CoA) is used in the subsequent Claisen condensation. It is one of the principal compounds present in *Zingiber officinale* with anti-inflammatory activity [27].

Tyrosol and hydroxytyrosol are phenolic alcohols present in olive oil together with oleuropein, a phenolic secoiridoid glycoside that consists of a hydroxytyrosol, elenolic acid, and a glucose molecule [28]. Oleuropein in particular has several health benefits as antioxidant, antimicrobial, antifungal, anti-tumoral, hypolipidaemic, hypotensive, and cardioprotective activities.

### 1.5. Analysis of Phenolic Compounds and Aim of this Review

Phenolic compounds are interesting natural products mainly for their antioxidant activity and usually, when performing an extraction from plant matrices, the antioxidant capacity is evaluated. This kind of measure is often intended as an evaluation of the total content of phenols. Phenolic compounds like phenolic acids, polyphenols, and flavonoids are able to scavenge free radicals such as peroxide, hydroperoxide, or lipid peroxyl. By this way they inhibit the oxidative mechanisms that lead to degenerative diseases and, for this reason, several assays have been proposed to determine the antioxidant capacity (AOC). Sometimes, the terms “antioxidant capacity” and “antioxidant activity” are used with the same meaning while sometimes they have different meaning as outlined in the recent review by Brainina et al. [29]. Despite this fact, the increasing number of papers reporting the determination of the AOC demonstrates the necessity to develop new systems to quickly measure this important parameter. The most common approaches reported in the literature regarding the AOC evaluation are relative methods since results for the same system are different. They are DPPH (2,2-diphenyl-1-picrylhydrazyl), ABTS-TEAC (2,2′-azino-*bis*(3-ethylbenzothiazoline-6-sulfonic acid- Trolox^®^ Equivalent antioxidant Capacity, ORAC (oxygen radical absorbance capacity), FRAP (ferric ion reducing antioxidant power), TRAP (total radical-trapping antioxidant parameter) and FC (Folin-Ciocalteau, for the determination of the total content of polyphenols) [30]. Although all these methods define the AOC of food components, they are based on different principles and use different ways of expressing the results, leading to incompatibility of values. Moreover, they are not selective for a single species. Conversely, the need for detection and quantification of specific compounds is increasing; for instance, the European Commission health claim on phenols in olive oil calls for the specific quantification of oleuropein and several related compounds such as tyrosol and hydroxytyrosol.

This review deals with all the classes of compounds reported above, and will discuss comprehensively significant examples of biosensors and sensing systems to detect them. Several recent reviews are partly related with this topic. In 2020, Ge, Li, and Lisak have reviewed sensing technologies for phenols in pharmaceutical and biomedical analysis [31]. The review deals with chemical sensors and biosensors. Among chemical systems, it reports colorimetric methods based on the general reactivity of phenols—as FC and related reactions—and advanced and very interesting approaches as the phenol-driven generation of Au nanoparticles. Electrochemical sensors are also reported while, among biosensors, the review discusses the use of several enzymes in optical and electrochemical systems. Conversely, the review does not cover biomimetic systems as those based on imprinted polymers, and other biological receptors for phenols. A further review in 2020 focuses on electrochemical biosensors for antioxidant activity [32]. It describes DNA- and enzyme-based electrochemical devices for the detection of antioxidant activity. A more specific review on the electrochemical detection of tea polyphenols also appeared this year [33]. In 2018, Della Pelle and Compagnone published a review on nanomaterials in sensing and biosensing of phenols and antioxidant activity [34]. The review focused on nanomaterials, including again metal nanoparticles generated by phenols in optical sensors, and quantum dots. Electrochemical systems involving nanomaterials and enzymes on nanostructured electrode surfaces were also reported. 

This review wishes to offer a complementary point of view, deriving from the scientific interests of our research group, which are not in the field of analytical chemistry. As organic chemists interested in natural products chemistry and in both bio- and biomimetic recognition of small molecules, we have identified two aims:

- To analyze the occurrence of specific recognition elements in sensing systems for phenolic compounds. To this end, we deal with biosensors and we include in this category also sensors based on designed, artificial biomimetic receptors as imprinted polymers. Such materials are in fact comprised in the definition of biosensors by the main journals, including this one. The question is: does the use of a specific recognition element lead to significant advantages in comparison to chemical sensors lacking designed complementarity although prepared with advanced and highly sophisticated materials? The main focus here is therefore on selectivity, obtained with, or without, the use of biological or biomimetic receptors, as enzymes, functional receptors, proteins, peptides, nucleic acids, and imprinted polymeric materials.
- To offer a comprehensive collection of data and references to the reader interested in the available methods for the detection of a specific compound. Here the focus is not only on selectivity and detectability, but rather on the ability of the sensors to operate in the real environment. Special attention is given to the systems whose analytical performance has been validated on real samples by comparison with a reference analytical methodology (most commonly a liquid chromatography-mass spectrometry method but not rarely also a capillary electrophoresis (CE) one), thus evaluating also matrix effects.

The review is organized first according to the sensing system, including electrochemical sensors (the large majority), optical and fluorimetric sensors, and other devices as gravimetric ones. Several tables summarize the main parameters of each sensor. In each table, sensors are grouped according to their target compounds, to allow the reader interested in biosensors toward a specific phenol to quickly find out the literature data on the required molecule. Brief discussions are reported at the end of each subsection, while the main discussion can be found at the end of the review. In the conclusive section, we try a comparison between systems including recognition elements and systems lacking them. The comparison includes the limits of detection attainable, the dynamic ranges, and selectivity. Future perspectives are given at the end.

## 2. Electrochemical Sensors

When dealing with analysis of phenolic compounds, electroanalytical methodologies are probably one of the most favorable options. Most of the biological activity of phenols is actually due to their ability to donate electrons to a wide set of receiver species that undergo reduction upon oxidation of the phenols. The redox potential of natural phenols and polyphenols spans over a quite large range and this represents a first source of selectivity that can in principle allow the multiple analysis of differently electroactive phenols by voltammetric techniques such as cyclic voltammetry (CV), differential pulse voltammetry (DPV), or square wave voltammetry (SWV). For instance, the peak potential of ten phenolic compounds found in white and red wine spans from 0.376 V of quercetin to 0.804 V of *p*-coumaric acid, including within this range important compounds such as gallic acid and caffeic acid, catechin, ferulic acid, resveratrol, malvidin, syringic acid, and vanillic acid [35]. Many electrochemical methods have been therefore developed to detect phenols even by simply using metal or carbon-based electrodes without attempts to enhance the selectivity by means of sensing elements, but rather to improve sensitivity by coatings of composite nanostructured materials, including mostly graphene, carbon nanotubes, and not-imprinted conducting polymers. Several significant examples are reported here below. The analytical performances of the electrochemical sensors reported in this review, as well as their key fabrication details, are reported in Table 1 and Table 2.

### 2.1. Electrochemical Sensors without Recognition Elements

#### 2.1.1. Carbon-Based Materials

In this section we report examples of “simple” carbon-based systems. Carbon materials have been widely explored for electrochemical sensing and offer several advantages: they are abundant, highly biocompatible, and span a wide range of physical and chemical properties which mainly depend on the carbon–carbon molecular orbital hybridization [36]. Among the most important carbon-based materials that have been applied to phenols sensing there are graphite, graphene, and carbon nanotubes as well as diamond-like materials, carbon black, and amorphous carbon, each one exhibiting unique properties.

Unmodified carbon graphite electrodes have been recently proposed by Søpstad et al. [37] for the detection of capsaicin at concentrations up to at least 5000 μM with a detection limit of 1.98 μM. The device was tested on real samples of chili-derived sauces available in Norwegian market and the values obtained were converted in SHU (Scoville Heat Units: a measure for pungency expressed as capsaicin equivalent content, currently obtained by chromatographic determination of capsaicin and dihydro capsaicin) which were in agreement with the ranges supplied by the manufacturers although no reference measurements were performed. 

Considering another phenolic compound, in 2019 Eremia and colleagues [38] developed a thick nano-crystalline graphite film on dielectric SiO_2_ substrate using plasma-enhanced chemical vapor deposition for the electrochemical detection of caffeic acid via chronoamperometry. The proposed method exhibited a limit of detection (LOD) of 43 μM and was tested on real matrices of chokeberries and berries, showing results that compared quite well with those obtained by high performance liquid chromatography –mass spectrometry (HPLC-MS) analysis. 

Graphene (GR) or reduced graphene oxide (rGO) is made up of two-dimensional one-atom-thick planar sheets of sp^2^-bonded carbon atoms arranged in a honeycomb structure [39], and it has attracted much attention as a superior electrode material for electrochemical sensing and biosensing applications owing to extraordinary electronic transfer properties, high electrical conductivity, high surface area, and good mechanical properties [40,41]. In 2014, Hu and coworkers [40] applied an electrochemically reduced graphene oxide (ERGO) modified glassy carbon electrode (GCE) to detect ferulic acid. Under the optimized conditions, the oxidation peak current was proportional to ferulic acid concentration in the range between 85 nM and 40 μM, with detection limit of 20 nM; the voltammetric sensor was also tested for detection of real content of ferulic acid in *A. sinensis* as well as in spiked biological samples of urine and blood, obtaining excellent recovery values. The measured contents were in agreement with those obtained by high performance liquid chromatography (HPLC). In 2015 Valentini and coworkers [42] developed two GR/ionic liquids nanocomposite gels—GR/bmim^+^Br^−^ and GR/bmim^+^Cl^−^ both having 1-butyl-3-methylimidazolium (bmim^+^) as cation and bromide and chloride as anion respectively; among the two assembled GR/paste electrodes, better analytical performances were observed in the case of the GR/bmim^+^Cl^−^ electrode, especially in terms of sensitivity per unit of area, reproducibility, fast response time, and LOD. The proposed electrodes proved to be very selective toward the oxidation of caffeic acid not only in presence of several common non-phenolic interferences, but also in presence of many polyphenols and flavonoid compounds, as it was assessed through permeability and perm-selectivity tests. Finally, satisfactory recovery values ranging from 97.2 to 99.7%, were obtained in real plasma, thus confirming the absence of matrix effects, and results were further confirmed by comparison with HPLC standard method. More recently, in 2019 Chen and coworkers [43] developed another electrochemical sensor for caffeic acid using a fluorine-doped GO modified GCE applied to real-time determination of caffeic acid in wine samples.

Other types of materials suitable for developing sensors for phenols detection comprise boron-doped nanodiamonds and amorphous carbon, owing to their high capability to prevent from passivation and fouling, and to their high sensitivities. Boron doped diamonds are composed of sp^3^ hybridized carbon electrodes in which boron, having one less electron than carbon, is introduced as charge acceptor to make the material conductive; on the other hand, amorphous nanocarbon is a mixture of sp^2^ and sp^3^ carbon atoms without a crystalline structure [36,44]. In 2018 Jiang et al. provided a comprehensive analysis of the electrochemical performance of three distinct carbon materials (graphene, nanodiamond, and nanocarbon) to detect monophenols (phenol and cresol) and biphenols (hydroquinone and catechol): the three carbon-based materials were converted into carbon nanoparticles which were drop-coated onto GCEs and were used to detect biphenols. GR and nanocarbon electrodes were also modified with tyrosinase, which facilitates monophenols detection by catalyzing their conversion into biphenols, whereas nanodiamond-modified electrodes could not be modified with the enzyme. Nanocarbon exhibited the lowest detection limit below 10 nM, and one order of magnitude higher sensitivity than the other carbon nanomaterials, whereas nanodiamond electrodes exhibited wider linear ranges and excellent anti fouling capability. The proposed electrodes were also used to detect co-existing phenol isomers in spiked river water and real green tea samples [36].

Carbon black (CB) is another interesting carbonaceous nanomaterial, its primary structure consists of spherical particles with diameters between 30 and 100 nm, and a secondary structure is formed by aggregates with sizes between 100 and 600 nm [34]. The main advantages of this carbon material are its excellent conductivity and electrocatalytic activity, as well as its cost-effectiveness. For example, Arduini et al. [45] developed in 2015 a sensor for phenols detection capable of selectively discriminating mono- and diphenols by modifying the surface of a screen-printed electrode (SPE) with a carbon black dispersion. The activity of the proposed CB-SPE was evaluated on catechol, gallic acid, caffeic acid, and tyrosol by SWV, and LODs were found to be in the range between 0.1 and 2 μM, exhibiting higher sensitivity and resistance to fouling problems at μM level, when compared with the bare SPE.

Carbon nanotubes are composed by hollow cylindrical tubes made up of carbon which have one or more concentric graphite layers capped by fullerenic hemispheres: they have been widely exploited in electrochemical sensing approaches as electrode materials owing to their unique structures and properties to provide strong electrocatalytic activity with minimal surface fouling [46]. Multi-walled carbon nanotubes (MWCNTs) were used in the electrochemical detection of capsaicin in 2008, when Compton et al. [47] outlined the voltammogram fingerprint of this molecule through adsorptive stripping voltammetry (AdSV) using a screen-printed carbon electrode modified with MWCNTs (MWCNT-SPEs) reaching a LOD of 0.45 μM. In 2016 Gao and coworkers [48] fabricated a MWCNTs-modified screen-printed electrode which was applied for electrochemical detection of chlorogenic acid through DPV in coffee beans, with recoveries ranging between 94.74 and 106.65%. The real content results were compared with those obtained by HPLC. The following year, Cheng and coworkers [49] fabricated another chlorgenic acid sensor based on chitosan (CS) and MWCNTs-modified GCE via a layer-by-layer self-assembly method. The proposed method showed an overall linear response to chlorgenic acid concentrations from 20 nM to 225 μM, and the LOD was estimated to be 10.6 nM. The sensor was tested for target molecule detection in biological samples, in particular human serum, using standard addition method, obtaining good recovery results that were validated by comparison with standard HPLC method.

A sensor for catechin determination was developed in 2019 by Şenocak et al. using single-walled carbon nanotubes (SWCNTs) covalently functionalized by terminal ethynyl bearing subphthalocyanine (SubPc) to obtain a new hybrid material, viz., SWCNT-SubPc via “click” reaction for the first time [50]. The results obtained were compared with SWCNT-modified GCE and bare GCE and it was shown that the deposition of SWCNT-SubPc on the surface of a GCE led to a 2.2 and 8-fold increase in the DPV responses to catechin in Britton-Robinson buffer solution (a pH of 3). Real samples of green, black, and fruit teas were analyzed, and the results obtained were compared with other catechin electrochemical sensors previously reported in the literature. As a main point, this sensor has a lower LOD (13 nM) and a wider dynamic range 0.1–1.5 μM compared to the majority of previously studied electrodes and it exhibited quite high stability and repeatability during the determination of catechin.

In summary, carbon-based materials have been effectively used to setup sensing systems for phenols. The detectability of targets is good, although not outstanding in most cases, and the median LOD value for the reported examples is 0.1 μM. The dynamic ranges are quite narrow. Some selectivity has been reported, as the ability to distinguish between mono- and diphenols. Fouling and electrode inactivation may represent a problem, but these phenomenons lower on carbon black and boron-doped diamonds.

#### 2.1.2. Composites of Carbon Materials and Inorganic Components

Carbon-based electrode materials are very often modified and combined with other functional materials, such as inorganic nanoparticles based for example on metals and metal oxides, in order to obtain composite materials exhibiting improved electrocatalytic activity if compared with electrodes based only on carbonaceous materials.

**Figure 5:**
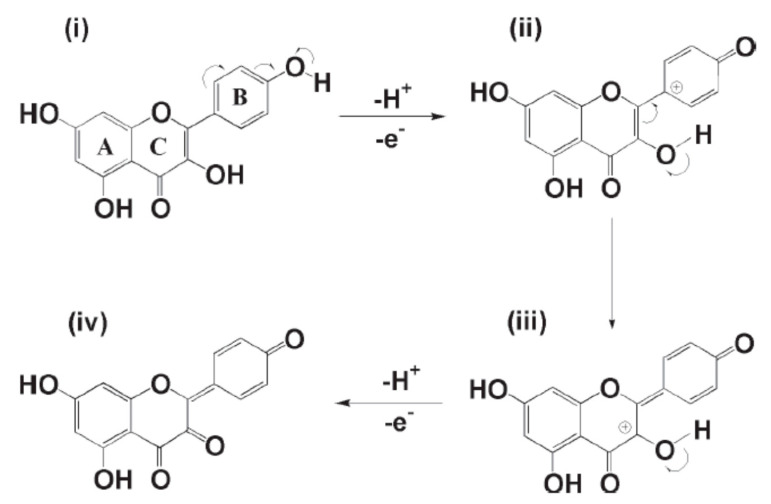
Mechanism of electrochemical oxidation of kaempferol at Fe_2_O_3_ NPs/MWCNTs/GCE. (**i**): structure of kaempferol; (**ii**): cationic intermediate after the first electron loss; (**iii**): charge delocalization of the cationic intermediate; (**iv**): final oxidation product after the second electron loss. Reproduced under the terms of the CC-BY Creative Commons attribution 4.0 from Reference [54], Copyright 2019.

**Figure 6:**
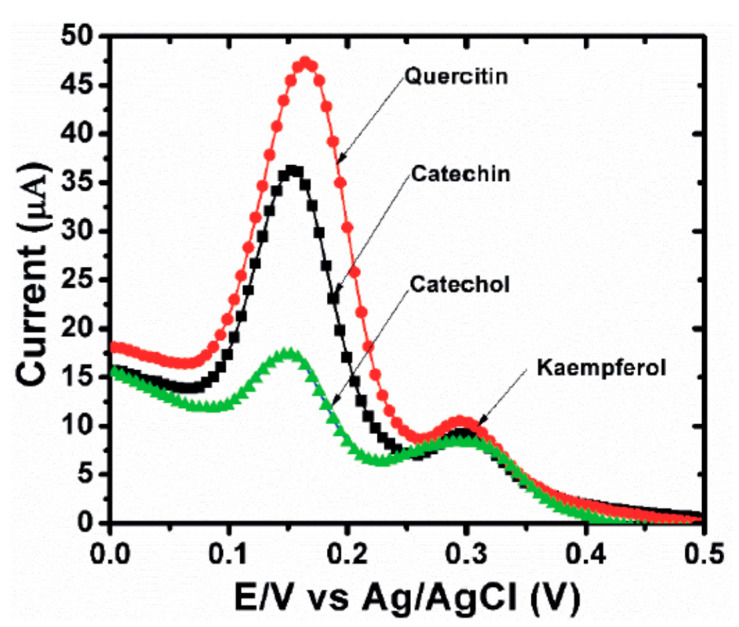
Effect of interfering compounds (quercetin, catechin, and catechol) on the detection of kaempferol under the optimum conditions. Reproduced under the terms of the CC-BY Creative Commons attribution 4.0 from Reference [54], Copyright 2019.

In 2015, catechin was determined using modified MWCNTs by Han et al. [51]. A GCE was modified with MnO_2_/carbon nanotubes decorated with a nanocomposite of Pt nanoparticles, and the electrochemical sensor exhibited a smaller peak potential separation (ΔEp), and faster electron transfer kinetics during the oxidation reaction of catechin. A very low detection limit of 0.02 μM for catechin was obtained with a linear range of 2–950 μM. Catechin was analyzed with the electrochemical sensor Pt/MnO_2_/f-MWCNT using SWV and the results were compared with those obtained with other electrodes, showing a lower LOD and a wider range of application. Real samples analysis of catechin in red wine, green tea, and black tea using the standard addition method revealed good practicability. Interferences with rutin, caffeine, gelatin, coumarin, quercetin, caffeic acid, hydroquinone, and catechol gave a signal change below 5% showing a good selectivity. More recently, in 2019 Karimi-Maleh and coworkers [52] developed a new electrochemical strategy based on the two-fold modification of carbon paste electrodes (CPE) with NiO-embedded SWCNTs nanocomposite and N-methyl-3-butylimidazolium bromide (CPE/MBIBr/NiO-SWCNTs) for the determination of ferulic acid in the presence of butylated hydroxytoluene, which was applied for the direct analysis of the two compounds in corn milk, wheat flour, and corn cider samples with good results. In 2020 the same group fabricated an ultrasensitive nano-molar voltammetric sensor for the determination of ferulic acid in the presence of sulfite in food samples by incorporating MgO/SWCNTs nanocomposite and 1-Butyl-3-methylimidazolium bis(trifluoromethylsulfonyl)imide [Bmim][Tf_2_N] into the carbon paste matrix to yield the modified CPE (MgO/SWCNTs-[Bmim][Tf_2_N]-CPE). The developed sensor had a wide linear response to ferulic acid concentrations in the range between 9 nM and 450 µM with LOD of 3.0 nM and was successfully applied for the determination of target molecule in real food matrices of red wine and white rice [53]. In 2019 Jiyane et al. developed an electrochemical sensor with a modified GCE for the detection of kaempferol by the immobilization of MWCNTs assimilated with Fe_2_O_3_ nanoparticles (NPs) onto the electrode surface using DPV. The proposed mechanism of oxidation of kaempferol is reported in Figure 5 [54]. Interferences from similar compounds, adding quercetin, catechin, or catechol independently to 0.50 g mL^−1^ of kaempferol, were also evaluated, but it was demonstrated that they hardly influence either the potential or the detection of kaempferol (Figure 6). The authors also made a comparison with previously reported electrochemical sensors, showing that this novel nanocomposite greatly enhances the anodic peak current of kaempferol, indicating a better effecting area. The range of applicability was found 1–300 μM with a LOD of 0.53 μM. The amount of kaempferol in broccoli real samples was determined to be 3.78 μg·g^−1^ but no other standard methods were applied to verify the accuracy of the data, although the result obtained was in line with literature data.

In 2019 Chen and coworkers developed a nanocatalyst based on copper sulfide nanodots grown on graphene oxide sheets nanocomposite (Cu_2_SNDs@GOS NC), synthesized by a simple sonochemical technique and applied it in electrocatalytic sensing of caffeic acid. The amperometric sensor exhibited a wide linear range covering five orders of magnitude between 55 nM and 2.46 mM, and the LOD was found to be 0.22 nM. After being successfully tested in the presence of 12 possible interfering compounds, the sensor was finally used to quantify the amount of caffeic acid present in carbonated soft drinks and red wine without sample pretreatments. The results were compared with those obtained by HPLC as reference method, showing very good accuracy of the proposed amperometric method [55]. In the last two years several electrochemical sensors have been proposed in the literature for the detection of quercetin. Quercetin was the targeted molecule in 2019 for different sensors using modified carbonaceous materials: the first one was developed by Mahdavi et al. [56] who fabricated a modified carbon paste electrode with zinc oxide dispersed on carbon nanosheet (ZnO/CNS/MCPE); the second one was developed by Fei et al. [57] who fabricated an electrochemical sensor based on mercapto-β-cyclodextrin functionalized reduced graphene oxide being noncovalently decorated by 1-pyrenebutyrate and gold nanoparticles (PBrGO/TCD/AuNPs). The best LOD was obtained with the second one, with a value of 1.83 nM in the linear range of 0.005-0.4 μM. The sensor showed also a good selectivity with respect to compounds with similar structures such as baicalin, galangin, resveratrol, morin and rutin. Analyses of real samples of apple juice, red wine and honeysuckle under optimum conditions were performed using the standard addition method and a recovery of 95–104.3% was obtained.

Considering graphene-inorganic composite materials, in 2016 Zhai and colleagues fabricated a GCE modified with gold microclusters (AuMCs) electrodeposited on sulfonate functionalized GR and applied the developed sensor for the simultaneous determination of gallic acid and uric acid. The anodic current was linearly related with concentrations ranging between 0.05 and 8.0 μM for gallic acid and 0.2 and 50.0 μM for uric acid, with the detection limits of 10.7 nM and 0.12 μM, respectively. The proposed sensor was also successfully applied for the determination of gallic acid in black tea and *Cortex moutan* as well as for the simultaneous determination of gallic acid and uric acid in urine samples, obtaining good recovery values and real gallic acid content results were validated by comparison with the standard HPLC method [41].

**Figure 7:**
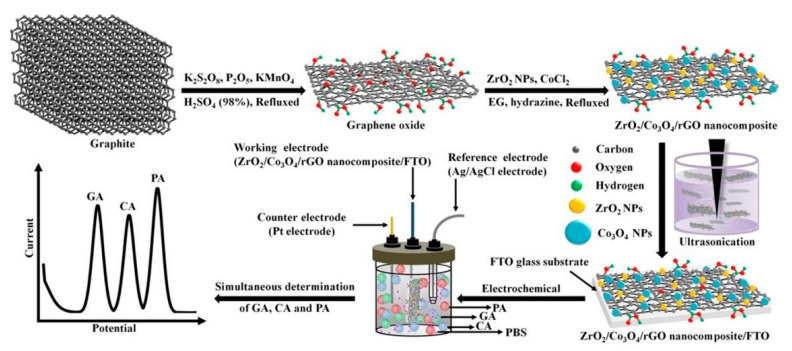
Fabrication of the ZrO_2_/Co_3_O_4_/rGO nanocomposite/fluorine-doped tin oxide (FTO) and scheme of the electrochemical apparatus for simultaneous detection of gallic acid, caffeic acid, and protocatechuic acid. Reprinted from ref. [39] with permission of Elsevier.

A very interesting electrochemical method for simultaneous detection of multiple phenolic compounds was proposed in the same year by Puangjan and coworkers [39], who developed a novel efficient ZrO_2_/Co_3_O_4_/rGO nanocomposite catalyst for the fabrication of an electrochemical sensor by casting it onto the surface of a fluorine-doped tin oxide (FTO) electrode (Figure 7). The ZrO_2_/Co_3_O_4_/rGO nanocomposite/FTO exhibited a synergistic catalytic effect toward the oxidation of gallic acid, caffeic acid, and protocatechuic acid with nanomolar limits of detection for each phenolic compound. Foreign compounds including phenolic and non-phenolic organic compounds were investigated as possible interferents and none of the tested molecules was found to interfere with the detection of the three target analytes. The developed sensor was applied for investigation of the three target compounds in real samples of fruit juice, tea, and rice by a standard addition method, giving good recovery values ranging between 95.4 and 101%.

Modified carbon ionic liquid electrodes (CILE) for the detection of luteolin have been proposed by Sun et al. in 2019 and 2018 [58,59] using platinum nanoparticles and biomass porous carbon (BPC) composite or gold nanocage. Both systems have been used for the determination of luteolin in Duyiwei capsules, and interferences of other flavonoids have been measured, like quercetin and baicalein. The Pt–BPC/CILE electrode was synthetized via a carbonization and activation process using wheat flour as the raw material for BPC, then, Pt–BPC nanocomposite was prepared via a hydrothermal method and further applied on the surface of CILE which was handmade by mixing 1-hexylpyridinium hexafluorophosphate (HPPF6) and graphite powder in a glass electrode tube. This system showed a lower LOD value (2.6 nM) and a wider linear range (0.008–100.0 μM) for luteolin detection with respect to other previously published methods. The analytical performances of the AuNCs/CILE-based electrochemical sensor using DPV were in the range 1–1000 nM with a LOD of 0.4 nM. Selectivity and recovery were good for both systems.

**Figure 8:**
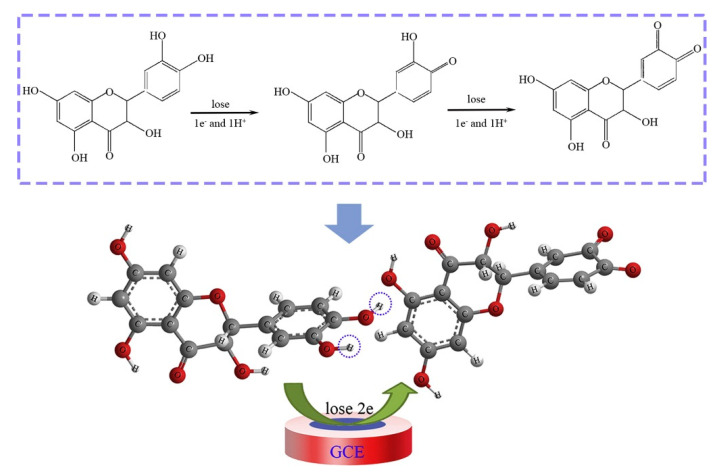
The redox reaction mechanism of taxifolin at MoS_2_/ANC/GCE. Reprinted from ref. [61] with permission of Elsevier.

In 2019, Guo et al. [60] developed an electrochemical sensor for luteolin based on CuCo coated nitrogen-enriched porous carbon polyhedron (Cu_1_Co_3_@NPCP) material. In particular, Cu_1_Co_3_@NPCP presented superior analytical performances as a lower detection limit (0.08 nM) in the linear range from 0.2 nM–2.5 μM, with an ultrahigh sensitivity as well as satisfactory reproducibility, and long-term stability. The sensor has been successfully utilized for determining luteolin in human serum samples using the standard addition method with satisfactory results, although interferences of other flavonoids were not considered. Xu et al. in 2020 [61] developed a MoS_2_ and activated nitrogen-doped active carbon composite glassy carbon electrode (MoS_2_/ANC/GCE) to detect taxifolin with very good performance using DPV. The linear range was 1 nM–1 μM with a low LOD (0.3 nM). The sensor showed stability and repeatability, and comparison with previously reported electrochemical sensors evidenced the wider range of application and the lower LOD obtained for this system. The selectivity was only measured using inorganic salts and ascorbic acid and glucose while it would be interesting to evaluate the selectivity toward other flavonoids which could be present in admixture. Extracts of fructus *polygoni orientalis* were used as real samples using the standard addition method to examine the practicability of the proposed method (Figure 8).

Always in 2019, Compagnone et al. [62] proposed a carbon black/molybdenum disulfide nanohybrid screen-printed electrode (SPE-CB/MoS_2_) to determine catechins in cocoa powder samples. The SPE-CB/MoS_2_ allowed an improvement of detectability (LOD ≤ 0.17 μM) of 100-folds compared to the bare screen-printed electrode, showing a linear range between 0.12 and 25 μM and the electrode was still active (recovery signal 99%) after measurements of 59 cocoa samples. DPV was used to quantify the analytes, and the best DPV conditions were found using the simplex method, with the final aim to maximize the signal/noise ratio. The applicability of the CB-SPE/MoS_2_ for the analysis of cocoa samples was determined by comparison with results obtained from the classical FC (total polyphenols) and ABTS (antioxidant capacity) assays as well as from AuNPs-based free-extraction approach, obtaining high correlation.

Considering electrodes based on metal oxides alone, in 2019 Yang et al. [63] developed an alumina microfiber-modified CPE for the detection of chlorgenic acid. The linear response of the proposed sensor to chlorgenic acid concentration ranged between 28 nM and 5.6 μM, and the LOD was evaluated to be 14 nM. The practical application of this new method was demonstrated by determining chlorgenic acid content in honeysuckle and soft drink samples by the standard addition method, and the accuracy of the proposed method was established by comparison with HPLC results taken as reference method. One year later, in 2020 Lin et al. [64] developed a simple and novel surfactant-free synthesis of flower-like strontium-doped nickel oxide nanorods via a simple sonochemical co-precipitation method to detect quercetin. The electrochemical detection of quercetin demonstrated a low detection potential of 0.3 V (vs. Ag/AgCl), and achieved a higher oxidation peak current compared to those of other modified electrodes in phosphate buffer (PB) (pH 5.0). The linear concentration range of application was 0.01–68.53 μM and the LOD was 1.98 nM. A comparison with several previously published methods was performed and analyses on real samples were made on apple and grape juice using the standard addition method.

Finally, detection of the isoflavone genistein has been very recently proposed by Chuanuwatanakul et al. [65] using a cobalt (II) phthalocyanine-modified screen-printed electrochemical sensor. The linear range was found to be 2.5–150 µM and the detection limit was 1.5 µM.

Such examples show clearly that the introduction of metal composite materials on carbon-based substrates leads to a significant improvement also in the detection of phenols. All the reported LODs are in the nanomolar range, with a median value of 2.6 nM, two orders of magnitude more favorable than with simple carbon-based materials.

#### 2.1.3. Polymeric Materials

Electrodes have also been modified with different types of polymeric materials exhibiting properties suitable for phenols sensing, very often in combination with carbon-based materials and, in some cases, also with the addition of other functional materials to obtain more complex composites.

In 2013 Filik and coworkers [66] developed an electrochemical sensor composed of Nafion–ERGO composite film grafted on a GCE, showing excellent electrocatalytic response to the oxidation of caffeic acid by square-wave adsorption stripping voltammetry, with a detection limit of 91 nM. The proposed sensor was tested against possible interfering compounds, exhibiting good anti-interference capability, and was successfully used to determine caffeic acid real content in diluted white wine samples, and results were validated through comparison with standard HPLC method.

Polymer-CNTs (carbon nanotubes) composite materials have attracted considerable interest due to their porous structure, large active surface structure as well as easy electron transfer ability with a synergistic effect that increases the catalytic activity of the surface [67]. Glassy carbon electrodes modified with multi-walled carbon nanotubes and electropolymerized gallic acid (poly(gallic acid)/MWNT/GCE) have been developed for direct quercetin quantification by Ziyatdinova et al. in 2018 [68]. A LOD of 54 nM was determined in the linear range of 0.075–100 μM for quercetin with a selectivity in the presence of the structurally related rutin. DPV was used and the results were compared with other polymeric film-modified electrodes showing a wider range of applicability. Quercetin quantification was performed in water and ethanolic extracts of medicinal herbs such as Bearberry leaves (*Arctostaphylos uva-ursi* (L.) Spreng.) and marigold flowers (*Calendula officinalis* L.). The oxidation peaks at 0.075–0.08 V observed on the DPVs of the extracts corresponded to the quercetin oxidation that had been proved by the standard addition method. The recovery of 97.6–101% confirmed the absence of the matrix effects. Moreover, validation for the quercetin contents in decoctions, infusions, and tincture has been obtained using a reference spectroscopic method. The following year, Karabiberoğlu and coworkers developed a composite electrode with poly L-methionine and MWCNTs onto a GCE for the determination of gallic acid. The prepared composite electrode exhibited in DPV a LOD of 3.1 nM, whereas the LOD determined through amperometry was found to be 0.5 nM. For validation purposes on real matrices, the developed electrode was applied to gallic acid determination in green tea, black tea, and red wine samples obtaining good recoveries; also gallic acid real content was determined, and results were in accordance with values obtained by LC-MS/MS, thus proving the accuracy of the proposed sensor [67].

Conductive polymers film have received much attention and are widely used in many electroanalytical applications due to their easy, fast, and controllable modification, strong adherence to the electrode surface, more active sites and good chemical stability [69,70].

In 2014 Chao and coworkers [70] fabricated through electropolymerization a poly-(aminosulfonic acid) modified GCE for the electrochemical determination of chlorgenic acid by CV. Under the optimized conditions, the oxidation peak current was linearly proportional to the concentration of chlorgenic acid in the range from 400 nM to 12 μM and the detection limit was found to be 40 nM. The proposed sensor was employed for real samples analysis on different traditional Chinese pharmaceutical products, and both recoveries of chlorgenic acid spiked samples and real content values were determined and validated through comparison with standard HPLC method.

In 2016, Yue and coworkers [71] fabricated bimetallic Pd–Au/PEDOT/rGO nanocomposites containing poly-3,4-ethylenedioxythiophene for electrochemical caffeic acid detection, showing a wide linear range of 1 nM–55 μM and LOD of 0.37 nM, as well as excellent selectivity; the proposed sensor was applied to caffeic acid detection in a real red wine sample with satisfactory recoveries ranging between 97.8 and 103.8%.

MWCNTs -modified GCEs were used for quantification of luteolin by Cheng et al. [72] and for luteolin and baicalein by Li et al. in 2020 [73]. Cheng used a nanomaterial composed of multi-walled carbon nanotubes, poly-3,4-ethylenedioxythiophene and gold nanoparticles (MWCNTs/PEDOT–Au) while Li used a modified GCE with poly(N-isopropylacrylamide-acrylic acid) hydrogel particles (NIPA/AA) and MWCNTs named NIPA/AA-MWCNTs-GCE. Both systems had excellent LOD for luteolin, resulting in 0.22 nM for the first system (linear range 0.001–15 μM) and 15 pM for the second system (linear range 0.0001–5 μM). Also for baicalein, an excellent LOD 44 pM was obtained in the linear range of 0.005–35 μM. To determine the selectivity of the two electrodes, MWCNTs/PEDOT–Au was used to detect luteolin in presence of the structurally similar compounds curcumin, quercetin, rutin, myricitrin, and diosmetin showing slight interferences with 10-fold concentrations of the other flavonoid compounds (signal change below 10%). MWCNTs/PEDOT–Au/GCE was tested for luteolin determination on human serum samples using SWV and the standard-addition technique while NIPA/AA-MWCNTs-GCE was used for simultaneous detection of luteolin and baicalein in real samples of peanut shell, traditional Chinese medicinal herb Huangqin and tomato.

Poly(*o*-phenylenediamine) has been exploited as a coating material showing selectivity toward rosmarinic acid and protocatechuic acid in 2019. Their amperometric detection gave micromolar ranges. The proposed sensor has been fully validated with HPLC reference in rosemary and melissa extracts, without interferences from caffeic acid, chlorgenic acid, *p*-coumaric acid, gallic acid, rutin [69].

In 2020, Mostafavi et al. [74] developed a polyaniline-based Fe_3_O_4_@SiO_2_-PANI-Au nanocomposite-modified glassy carbon electrode for quercetin detection in the concentration range from 0.01 μM to 15 μM, and with a LOD of 3.8 nM. Biological (human serum and urine) samples, tea, radish leaves, and apple juice samples were spiked with 10 nM or 5 μM concentration levels of quercetin and analyzed by DPV and compared to the HPLC results. Good recovery percentages were obtained as well as a good accordance with the HPLC standard method results. Interferences were not considered with other flavonoids, but only with compounds such as glucose, uric acid, caffeine, and ascorbic acid showing a slight interference with the targeted analyte.

Poly (diallyldimethylammonium) chloride (PDDA) is a linear positively charged polyelectrolyte which was introduced because the functional groups and the non-covalent interactions of polymer with the GR surface maintain the electrical property of GR and also improve its dispersibility [75]. A voltammetric graphene-based sensor for gallic acid was proposed in 2016 by Li and coworkers by using the poly(diallyldimethylammonium chloride)-functionalized GR supported platinum nanoparticles nanocomposite (PDDA-GR-Pt) modified GCE. Detection of gallic acid was conducted by SWV, with linear concentration range between 30 nM–1 μM and limit of detection of 7 nM. The proposed sensor was also tested on real matrices of *Jianmin Yanhou* tablets, *Cortex moutan* and green tea, obtaining excellent recoveries in the range of 99.8–102.3%. The real content of gallic acid in the real matrices was also validated by comparison with HPLC reference method [76]. The following year Ye et al. [75] proposed a modified graphene electrode using PDDA to improve the dispersibility of graphene and the metal oxide SnO_2_ to obtain the SnO_2_-PDDA-GR composite which exhibited high specific voltammetric response to daidzein. The developed electrochemical sensor showed a LOD of 6.7 nM in the linear range 0.02–1 μM. The results obtained for daidzein determination were compared with the already known electrochemical sensors and the analyses on real samples of *Pueraria lobata* and commercial daidzein tablets were validated with HPLC showing a good correspondence.

In summary, the introduction of polymeric materials seems to allow a further improvement in the detection of phenols. LODs are nano- or subnanomolar, with a 500 pM median value. Moreover, most of the papers in this subsection discuss also the performance of the sensors in real samples, and the effects of real matrices. This clearly indicates that polymers allow to operate in complex environments with reduced drawbacks from the presence of contaminants.

#### 2.1.4. Metal Organic Frameworks and Covalent Organic Frameworks

As an emerging class of crystalline porous materials, covalent organic frameworks (COFs) that are derived from light elements linked by strong covalent bonds through the principles of the reticular chemistry, have received great attention in the past decade owing to interesting properties such as their structural periodicity, mechanical robustness, and uniform porosity [77]. In 2018 Wang and coworkers [77] fabricated a novel gold nanoparticles-doped TAPB-DMTP-COFs composite (TAPB is 1,3,5-*tris*(4-aminophenyl)benzene; DMTP is 2,5-dimethoxyterephaldehyde) via covalent organic frameworks as the host matrix to support the growth of gold nanoparticles. The novel composite was used to constitute a TAPB-DMTP-COFs/AuNPs-modified GCE for electrochemical detection of chlorgenic acid, exhibiting a wide linear range of 10 nM–40 μM, and a low LOD of 9.5 nM. The sensor exhibited excellent selectivity with many possible interfering compounds, including many phenols, and was applied to determine target molecule content in real matrices of apple, coffee and honeysuckle, obtaining excellent recoveries ranging from 99.2 to 102.5% and real content results were validated by comparison with standard HPLC.

Metal-organic frameworks (MOFs) are hybrid inorganic–organic microporous crystalline materials which have aroused great attention for electrochemical sensing applications owing to many favorable properties such as their tunable morphology and large specific surface area. However, their use is limited by their poor conductivity and instability [78,79]. In order to overcome these problems, a favorable possibility is that of integrating two components of functional materials like MOFs and conductive materials in order to combine their complementary properties [79]. In 2016 Wang and coworkers [80] synthesized an electrochemical sensor based on metal–organic frameworks/titanium dioxide nanocomposites for the detection of chlorgenic acid. The proposed sensor exhibited a linear response to chlorgenic acid concentrations in the range of 10 nM–15 μM and a LOD of 7 nM. The sensor exhibited excellent selectivity toward many possible interfering compounds and was successfully applied for the detection of chlorgenic acid in real matrices of coffee and tea samples.

Very recently, a sensor for quercetin and luteolin was proposed by Wu et al. [81] who synthetized three kinds of erbium-based metal-organic frameworks with different morphologies using erbium nitrate and 1,3,5-benzenetricarboxylic acid (H_3_BTC) as the source, by adding various amounts of ammonium acetate. The prepared Er-BTC had different morphologies and different signal enhancement ability toward the oxidation of quercetin and luteolin, reaching a LOD of 0.22 nM for quercetin and 0.14 nM for luteolin in the linear range 0.5–100 nM for quercetin and 0.5–80 nM for luteolin. The new sensor was applied to the analysis of drink and tea samples, and the results were in good agreement with those obtained from a standard HPLC method with a relative error below 5%. Simultaneous sensing of quercetin and luteolin is possible since the oxidation of quercetin and luteolin on carbon paste electrode (CPE) modified with Er-BTC-3 are independent with weak mutual interference.

In the same year, a core-shell GR/CuO@Cu-(1,3,5-benzene-tricarboxylate) composite (GR/CuO@Cu-BTC) for electrochemical sensing of caffeic acid was synthesized by using flowerlike CuO/GR as substrate template and Cu^2+^ source for the formation of Cu-BTC shell. The sensor was applied to the voltammetric determination of caffeic acid in wine, giving results in agreement with those obtained through HPLC. It exhibited a good linear response to caffeic acid concentration in the range between 20 nM and 10 µM, together with a low LOD of 7.0 nM [78].

Wang et al. [82] developed in 2019 a novel core-shell structure composite with traditional conducting polymer polypyrrole (PPy) as its core and zeolitic imidazolate framework-8 (ZIF-8) shell structure for quercetin detection in human plasma samples.

In 2015 Lu et al. employed for the first time mesoporous cellular foams (MCFs) modified carbon paste electrode (MCFs/CPE) for the determination of capsaicin [83]. The proposed modified electrode showed high sensitivity toward the oxidation of capsaicin in the linear range of 0.76–11.65 µM with a LOD of 0.08 µM and the results obtained on hot pepper samples were in good agreement with the HPLC standard analyses. Interferences with catechol and *p*-chlorophenol as well as inorganic salts were evaluated and the current responses were negligible, thus demonstrating that no interference occurred. The extracted solutions were deluted in 0.1M perchloric acid (pH 1.0) and the DPV curves were recorded from 0.1 to 1.1 V after 60 s accumulation at 0.25 V.

In conclusion, MOFs and COFs perform similarly to polymeric materials in the analysis of phenols, with nanomolar LODs and reduced matrix effects. Selective systems have been obtained with these techniques.

#### 2.1.5. Photoelectrochemical Systems

Photoelectrochemical sensors are considered a novel type of analytical devices based on the photoelectrochemical properties of semiconductor materials; among the advantages of this type of sensors, which are based on the interactions between analyte and a photoactive material, there are their ease and low-cost instrumentation, their sensitivity and low noise due to the different principles involved in excitation and detection [79]. In 2017, Han and coworkers fabricated a novel photoelectrochemical sensor for the selective analysis of gallic acid based on Bi_2_S_3_ accommodated in Bi_2_MoO_6_ nanobelts, which conferred a platform with excellent light-harvesting capability, selectivity and reproducibility [84]. Bi_2_MoO_6_/Bi_2_S_3_ exhibited the best capability to distinguish between gallic acid and other antioxidant compounds, also showing good anti-interferences capability, and was thus applied for estimating gallic acid content in real drug samples, giving real content results perfectly coherent with those obtained through HPLC. A few years later, Damos and coworkers proposed another selective sensor targeting caffeic acid, based on titanium dioxide nanoparticles (TiO_2_), carbon nanotubes (CNTs), and cadmium telluride quantum dots (CdTeQDs) on a fluorine-doped tin oxide electrode [79]. Under optimized experimental conditions, the photocurrent of the sensor showed a linear relationship with the increase of the caffeic acid concentration from 0.5 to 360 μM and a LOD of 0.15 μM, and the proposed method was applied for the determination of caffeic acid in samples of soluble coffee and tea obtaining good recovery values of 99.9% and 97.4% respectively for the two tested real samples.

In 2020, the same group proposed a photoelectrochemical sensor for the determination of naringin at a zero-biased fluorine-doped tin oxide electrode modified with cadmium sulfide and titanium dioxide, sensitized with chloroprotoporphyrin IX iron(III) using a 20W LED lamp as irradiation source [85]. The sensor had a LOD of 0.03 μM in the linear response range from 1 to 332 μM at low potential (0 V vs. Ag/AgCl). The new sensor results were compared with other electrochemical sensors previously developed for the determination of naringin resulting in a good LOD and a wide range of linearity. Real samples of citrus fruit juice were analyzed and interference with other organic compounds such as ascorbic acid, hesperidin, naringenin, flavone, gallic acid, quercetin, citric acid, fructose, glucose, and inorganic salts was also evaluated. It was shown that ascorbic acid and calcium can interfere of 15 and 12.5% on the naringin response.

**Table 1:** Performance of electrochemical systems without recognition elements.

Sensing System (Electrochemical Technique)	Target	LOD	Linear Range	Non Interfering Related Compounds (Interfering Related Compounds)	Recovery	Reference Method	Real Samples (Sample Preparation)	Ref.
SWCNT-SubPc (DPV)	catechin	13 nM	0.1–1.5 μM	rutin, 6-methoxy flavone, gallic acid, caffeic acid	96.5–98%	no	green, rosehip fruit, Turkish and Indian black tea (EtOH ext.)	[50]
Pt/MnO_2_/f-MWCNT/GCE (CV)	catechin	0.02 μM	2–950 μM	rutin, quercetin, caffeic acid, catechol	99.2–101%	no	red wine, black tea, and green tea samples (not reported)	[51]
Nanocarbon-GCE, Nanodiamond-GCE, Graphene-GCE (CV, SWV, Amp)	catechol, hydroquinone, cresol, phenol	0.04–0.11 μM (nanocarbon); 0.10–0.2 μM (graphene), 0.12–0.43 μM (nanodiamond)	Up to 100 μM hydroquinone (nanodiamond)	Nanocarbon electrode distinguishes catechol and hydroquinone	101% (catechol) and 102% (hydroquinone) (nanocarbon, river water)	no	river water (nanocarbon electrode: no pretreatment requested), tea (water inf.)	[36]
Nafion/ER-GO/GCE (SW-AdSV)	caffeic acid	0.091 μM	0.1–10 μM	*p*-coumaric acid, sinapic acid, ferulic acid, gallic acid, catechin (chlorgenic acid at same concentration as caffeic acid caused a positive interference of 14%)	97–98%	HPLC	white wine (dil.)	[66]
GR/bmim^+^Br^−^ PE and GR/bmim^+^Cl^−^PE (DPV)	caffeic acid	5 μM (G/bmim^+^Cl^−^PE) and 18 μM (G/bmim^+^Br^−^PE)	0.025–2.00 mM	Many polyphenols and flavonoids compounds (permeability and perm-selectivity test)	97.2–99.7%	HPLC on spiked plasma samples	human plasma (no pretreatment requested)	[42]
Pd–Au/PEDOT/rGO/GCE (DPV)	caffeic acid	0.37 nM	0.001–55 μM	catechol, *p*-coumaric acid, gallic acid, vanillic acid, sinapic acid, ferulic acid	97.8–103.8%	no	red wine (dil.)	[71]
GR/CuO@Cu-BTC/GCE (DPV)	caffeic acid	7.0 nM	0.020–10.0 μM,	catechol, lemon yellow	95.91–104.60% calculated based on HPLC values	HPLC	red wine (dil.)	[78]
Cu_2_S NDs@GOS NC/SPCE (Amp)	caffeic acid	0.22 nM	0.055–2455 μM	gallic acid, dopamine, hydroquinone, *catechol*, epinephrine, norepinephrine, resorcinol	96.18–99.43% calculated based on HPLC values	HPLC	red wine, soft drinks (dil.)	[55]
F-GO/GCE (DPV)	caffeic acid	0.018 μM	0.5–100.0 μM	*p*-coumaric acid, hydroquinone, ferulic acid, gallic acid	n.d.	1 sample of 4 compared with HPLC result	wine (no pretreatment requested)	[43]
NCG electrode (ChAmp)	caffeic acid	43 μM	50 μM–1mM	results not shown for interferences studies performed on matrix	n.d.	HPLC-MS	berries and chokeberries (MeOH + 2% formic acid ext.)	[38]
CB-SPE (SWV)	caffeic acid, catechol, gallic acid, tyrosol	0.1 μM (catechol), 0.8 μM (caffeic acid) 1 μM (gallic acid), and 2 μM (tyrosol)	1–50 μM (catechol), 1–50 μM (caffeic acid), 10–100 μM (gallic acid), 10–100 μM (tyrosol)	Tested amongst target molecules: the sensor distinguishes mono and diphenols	n.d.	no	no	[45]
TiO_2_/CNTs/CdTeQDs/FTO (photoelectrochemistry)	caffeic acid	0.15 μM	0.5–360 μM	chlorgenic acid, gallic acid, vanillic acid, ferulic acid, quercetin, caffeic ethyl ester	96.2–101.3%,	no	soluble coffee (water dispersion) and tea sachets (water inf.)	[79]
SPE-CB/MoS_2_ (DPV)	catechins	LOD ≤ 0.17 μM	0.12–25 μM	n.d.	94–103%	Folin Ciocalteu, ABTS	cocoa (DMSO ext.)	[62]
MWCNTs/SPE (CV, DPV)	chlorgenic acid	0.34 μM	0.48 μM–45 μM	n.d.	94.74–106.65%	HPLC	coffee beans (hexane 1:6 *w/v* in Soxhlet extraction system + water microwave assisted ext.)	[48]
(CS/MWCNTs)_6_/GCE (DPV)	chlorgenic acid	11.6 nM	0.02–225 μM	other compounds not similar to the target	99.33–109.0%	HPLC (based on standard addition method)	human serum (dil.)	[49]
PASA/GCE (CV)	chlorgenic acid	40 nM	0.4 μM–12 μM	other compounds not similar to the target	96.3–102.8%	HPLC	pharmaceutical products (no pretreatment requested) and honeysuckle (EtOH ext. + dil.)	[70]
TAPB-DMTP-COFs/AuNPs/GCE (DPV)	chlorgenic acid	9.5 nM	10 nM–40 μM	dopamine, L-dopa, hydroquinone, catechol, thymol, rutin, quercetin, caffeic acid, gallic acid, vanillic acid	99.2–102.5%,	HPLC	coffee, apple, honeysuckle (not reported)	[77]
alumina microfiber-modified CPE (DPV)	chlorgenic acid	14 nM	28 nM and 5.6 μM	other compounds not similar to the target	n.d.	HPLC	honeysuckle (EtOH ext.) and soft drinks (fil.)	[63]
MOF/TiO_2_/GCE (DPV)	chlorgenic acid	7 nM	0.01–15 μM	catechol, dopamine, hydroquinone, paracetamol, caffeic acid, rutin, ferulic acid, gallic acid, vanillic acid	96.0–102.0%	no	coffee and tea (not reported)	[80]
SPE (coarsely stepped cyclic SWV)	capsaicin	1.98 μM	0–5000 μM	n.d.	n.d.	no	chili derived sauces (EtOH ext.)	[37]
MWCNT-BPPGE and (MWCNT-SPE)	capsaicin	0.31 μM (0.45 μM)	0.5–60 mM (0.5–35mM)	n.d.	n.d.	correlation with the average Scoville unit values reported in the literature for these sauces	hot pepper sauces (EtOH ext.)	[47]
MCFs/CPE (DPV)	capsaicin	0.08 μM	0.76–11.65 μM	catechol, *p*-chlorophenol	96–101.1%	HPLC	hot pepper powder	[83]
SnO_2_-PDDA-GR/GCE (LSV)	daidzein	6.7 nM	0.02−1.0 μM	other compounds not similar to the target	97.81–103.30%	HPLC	pueraria lobata (EtOH ext.)	[75]
ERGO/GCE (DPV)	ferulic acid	20.6 nM	0.085–38.9 μM	other compounds not similar to the target	99.77–101.73% (*A.sinensis*); 95.72–104.51% (biological samples)	HPLC (*A. sinensis*)	*A. sinensis* (70% EtOH reflux ext. + dil.), urine and plasma (dil.)	[40]
CPE/MBIBr/NiO-SWCNTs (SWV)	ferulic acid (and butylated hydroxytoluene)	20.0 nM (ferulic acid)	0.06–900.0 μM (ferulic acid)	other compounds not similar to the target	n.d.	HPLC	corn milk (hot water), wheat flour (sulfuric acid dissolution + hot water ext.), corn cider (fil.)	[52]
MgO/SWCNTs-[Bmim][Tf_2_N]-CPE (DPV)	ferulic acid (and sulfite)	3.0 nM	0.009–450 μM	other compounds not similar to the target	99.17–101.6% (ferulic acid)	HPLC	red wine (fil.) and white rice (50% EtOH+ sulfuric acid dissolution)	[53]
ZrO_2_/Co_3_O_4_/rGO nanocomposite/FTO (DPV)	gallic acid, caffeic acid, protocatechuic acid	1.56 nM (gallic acid), 0.62 nM (caffeic acid), 1.35 nM (protocatechuic acid)	6.24–477.68 nM (gallic acid); 2.48–524.90 nM (caffeic acid); 5.40–424.96 nM (protocatechuic acid)	gentisic acid, sinapic acid, vanillin, *p*-coumaric acid, vanillic acid, *p*-hydroxybenzoic acid, vitamin B1, vitamin B2, morin hydrated, rutin, ellagic acid	95.4–100% (gallic acid); 96.1–99.5% (caffeic acid), 95.5–101.0% (protocatechuic acid)	no	fruit juice (fil. + dil.), rice (85% MeOH ext. + dil.) and tea samples (inf.+fil. +dil.)	[39]
PDDA-GR-Pt/GCE (SWV)	gallic acid	7 nM	0.03–1 μM	other compounds not similar to the target	99.8–102.3%	HPLC	Jianmin Yanhou tablets (MeOH ext.), Cortex moutan (MeOH ext. + dil.) and green tea beverage (no pretreatment requested)	[76]
PLM/MWCNT/GCE (DPV, Amp)	gallic acid	3.1 nM (DPV) and 0.5 nM (Amp)	DPV: (4.0 nM–20.0 μM); Amp: (2.0 nM–12.0 μM)	(100 times higher concentrations of caffeic acid, gallic acid oxidation peak current was increased about 22%.)	96.74–101.49%	LC-MS/MS	black and green tea (inf.+ dil.), red wine (dil.)	[67]
heterostructured Bi_2_MoO_6_/Bi_2_S_3_ nanobelts (photoelectrochemistry)	gallic acid	n.d.	24.88–348.84 μM	discrimination of gallic acid from other antioxidant compounds (+)-Catechin hydrate, caffeic acid, chlorgenic acid, (-)-Epicatechin, myricetin	99.58%–101.37%	HPLC	rose oral liquid and pomegranate enrich blood syrup (not reported)	[84]
AuMCs/SF-GR/GCE (DPV)	gallic acid (uric acid)	10.7 nM (0.12 μM uric acid)	0.05–8.0 μM gallic acid (0.2–50.0 μM uric acid)	other compounds not similar to the target (some polyphenolic compounds with ortho-diphenol groups at B-ring could interfere)	96.0–102.4% (gallic acid) (97.0–102.4% UA) in urine; 96–100.8% in black tea and *Cortex moutan*	HPLC	urine (dil.), *Cortex moutan* (MeOH ext.), black tea (inf. + dil.)	[41]
CoPC modified SPCE (SWV)	genistein	1.5 µM	2.5–150 µM	other compounds not similar to the target	99.98-104.68%	no	Derris scandens extracts (EtOH ext.)	[65]
Fe_2_ O_3_ NPs/MWCNTs/GCE (DPV)	kaempferol	0.53 μM	1–300 μM	quercetin, catechin, CC	99.55% average	no	broccoli (EtOH ext.)	[54]
Pt-BPC/CILE (CV, DPV)	luteolin	2.6 nM	0.008–100.0 μM	quercetin, baicatin, rutin	98.33–103.75%	no	Duyiwei capsule (EtOH ext.)	[58]
AuNCs/CILE (DPV)	luteolin	0.4 nM	1–1000 nM	quercetin, baicalein	95.0–96.7%	no	Duyiwei capsules (EtOH ext.)	[59]
Cu_1_Co_3_ @ NPCP	luteolin	0.08 nM	0.2 nM to 2.5 μM,	other compounds not similar to the target	99.6–102.2%	no	human serum samples (not reported)	[60]
MWCNTs/PEDOT–Au/GCE (CV, SWV)	luteolin	0.22 nM	0.001–15 μM	curcumin, quercetin, rutin, myricitrin, diosmetin	99–103%	no	human serum samples (not reported)	[72]
NIPA/AA-MWCNTs-GCE (DPV)	luteolin/baicalein	0.0145 nM/0.0444 nM	0.0001–1.5 mM/0.005–35 mM	other compounds not similar to the target	93.3–106.6%	no	peanuts shell, tomato (EtOH ext.)	[73]
CPPI-TiO_2_/CdS/FTO (photoelectrochemistry)	naringin	0.03 μM	1–332 μM	hesperidin, flavone, gallic acid, quercetin, naringenin	97.8–99.6%	no	orange, lemon, tangerine juice (dil.)	[85]
SNO NRs/GCE	quercetin	1.98 nM	0.01–68.53 μM	rutin	86–99.6%	no	apple and grape juice (dil.)	[64]
Fe_3_O @ SiO_2_-PANI-Au nanocomposite/GCE	quercetin	3.8 nM	0.01–15 μM	other compounds not similar to the target	96–102%	HPLC	human serum and urine samples, tea, radish leaves, and apple juice samples (not reported)	[74]
ZnO/CNS/MCPE (DPV)	quercetin	0.04 μM	0.166–3.63 μM	rutin	90.8–113.0%	no	onion and honey buckwheat (dil. PBS)	[56]
PB-rGO/TCD/AuNPs (CV, DPV)	quercetin	1.83 nM	0.005–0.4 μM	morin, galangin, resveratrol, baicalin, rutin	95–104.3%	no	apple juice, red wine and honeysuckle (fil. + dil.)	[57]
PPy @ ZIF-8 (DPV)	quercetin	7 nM	0.01–150 μM	hyperin, delphindin, catechin hydrate; rutin, luteolin, kaempferol	99.1–102.6%	no	human blood plasma (dil. + PBS + ACN)	[82]
poly(gallic acid)/MWCNT/GCE (DPV)	quercetin	54 nM	0.075–100 μM	rutin, vanillin, syringaldehyde, gallic acid, ferulic acid, *p*-coumaric acid, sinapic acid	97.6–101%	UV	medicinal herbs extract (water inf. or dec.)	[68]
Er-BTC	quercetin/luteolin	0.22 nM/0.14 nM	0.5–100 nM/0.5–80 nM	other compounds not similar to the target	n.d.	HPLC	drink and tea samples (fil. + dil.)	[81]
GCE/PoPD/Pt (DPV and ChAmp)	rosmarinic acid, protocatechuic acid	ChAmp: 0.5 μM (rosmarinic acid) and 0.6 μM (protocatechuic acid); DPV: 0.7 μM (rosmarinic acid, protocatechuic acid)	ChAmp: 1–55 μM (rosmarinic acid) and 1–60 μM (protocatechuic acid); DPV: 2–10 μM (rosmarinic acid) and 1–35 μM	caffeic acid, *p*-coumaric acid, chlorgenic acid, gallic acid, 2,5-dihydroxybenzoic acid, rutin	n.d.	HPLC	rosemary and melissa extracts (water inf.)	[69]
MoS_2_/ANC/GCE (DPV)	taxifolin	0.3 nM	1 nM–1 μM	n.d.	98.9–100.5%	no	fructus polygoni orientalis (MeOH ext.)	[61]

Dil.: dilution; Fil.: filtration; ext.: extraction; inf.: infusion, dec.: decoction.

### 2.2. Electrochemical Sensors Equipped with Recognition Elements

We focus here on selectivity obtained, or improved, by exploiting specific recognition elements grafted onto the electrochemical sensors.

#### 2.2.1. Enzymes

Oxidizing enzymes are used in electrochemical detection of phenols owing to their ability to oxidize such compounds with different mechanisms and to yield different products, either quinones deriving from simple oxidation, or even compounds containing further oxygen. Their catalytic role allows amplification of the electrochemical signals obtained by back reduction of the oxidation products of the phenolic analytes. They can be therefore regarded as functional components of the electrochemical sensor. However, the recognition process of their substrates operates on the basis of their action. They are thus also recognition elements for the target molecules, and their substrate selectivity leads to the detection of classes of phenols or specific molecules.

Their use on the electrode surfaces is not novel, but calls for the resolution of typical problems, arising from the need of correct immobilization, compatibility with several of the advanced materials listed in the previous section, changes in the conductive properties of the surface, inactivation by inhibition at the catalytic site level.

##### Tyrosinase

Tyrosinase (monophenol monoxygenase) is a copper enzyme that can accept monophenols as substrates to catalyze their oxidation to *o*-diphenols by molecular oxygen, and then the further oxidation of *o*-diphenols to *o*-quinones. These end-products are then detected electrochemically following their reduction back to *o*-diphenols with relatively high sensitivity at low potentials. The selectivity of tyrosinase—based electrochemical sensors has been studied by Adreescu and Sadik [86] on phytoestrogens (resveratrol, genistein and quercetin) and synthetic estrogens (diethylstilbestrol, nonylphenol, bisphenol A), in a study aimed at developing methods to monitor this kind of endocrine-disrupting compounds that are strongly related to the occurrence of hormone-induced cancers and reproductive disorders in humans and wildlife as well. The enzyme was included directly inside the carbon paste of the working electrode to obtain the biosensor. Real samples containing binary or tertiary mixtures of the estrogens were analyzed, and the biosensor results were compared with a reference spectrophotometric method. The sensor was able to detect resveratrol, genistein, and bisphenol A with LODs in the 100–150 µM range, while diethylstilbestrol and nonylphenol resulted not to be substrates for the immobilized enzyme. Rather than directly related to the enzyme selectivity, the authors propose that the accessibility of the catalytic site in the carbon paste is important to the performance, and this disfavors the bulkiest compounds.

Tyrosinase was used in 2013 by Calas-Blanchard et al. to develop an amperometric biosensor to quantify several catechin derivatives frequently found in teas [87]. The enzyme was immobilized by coreticulation with glutaraldehyde on SPEs leading to results that were in good agreement with the HPLC method of analysis. Since tyrosinase catalyzes the oxidation of *o*-diphenols (including catechins) to *o*-quinones by consumption of molecular oxygen, this reaction can be successfully applied to the determination of phenolic compounds as the quinones formed are electrochemically reduced at a low potential and the measured current is proportional to the phenolic compound concentration. The sensor was first applied to the determination of catechol, with a detection limit of 0.03 μM, lower than the values already reported in literature for other tyrosinase biosensors. Subsequently it was applied for the determination of catechin derivatives, finding the following trend catechol >>>> epicatechin > catechin > epicatechin-3-gallate > gallocatechin >epigallocatechin > epigallocatechin-3-gallate which demonstrates the much higher affinity of tyrosinase for catechol of 4 to 128 times with respect to the catechin derivatives. The SPE/Tyr sensor was so used for the determination of the phenolic content of tea samples expressed both in catechin equivalent phenolic content (catechin-EPC) and epigallocatechin gallate equivalent phenolic content (epigallocatechin-3-gallate-EPC). In the meanwhile, the HPLC analyses of seven catechins derivatives were also performed on green and black teas samples showing a good correlation.

##### Laccases

Laccases are also multicopper oxidases, they catalyze single-electron transfer oxidation of phenolic derivatives leading to reactive intermediates that can then further react to cross-linked products (this may represent a drawback due to the potential inactivation of the biosensor by deposition of insoluble products). Laccases are also involved in the degradation of lignins and in the synthesis of melanin. To develop a resveratrol biosensor, laccase from *Coriolus Versicolor* has been immobilized on derivatized polyethersulphone membranes and applied to a Pt-Ag, AgCl electrode base [88]. The sensor was capable to detect resveratrol with a 1 µM LOD. The sensor is thus more sensitive to resveratrol than the above reported tyrosinase one. However, the linear range is even narrower. Its selectivity was good when tested in model solutions on caffeic acid, gallic acid, catechin, rutin, quercetin and malvidin, but this result could not be transferred to real samples of red wine due to strong matrix effects, and pretreatment by solid phase extraction was in the end needed to exploit the sensor.

A further interesting example of laccase-based biosensor was described for the detection of caffeic acid, rosmarinic acid, and gallic acid [89]. In this case, laccase was coupled with polydopamine, an emerging adhesive bioinspired material that mimics the adhesion mechanism of mussels. The polymeric material was synthesized directly on the surface of glassy carbon or graphite electrodes by electropolymerization carried out in the presence of laccase. The mechanism of oxidation of the three target acids was studied in deep detail, and then an analytical methodology was set up. Submicromolar LODs were obtained, with rosmarinic acid detected at 90 nM level (Table 2). A particularly wide range of linear detection was observed for gallic acid (1–150 µM). The sensor was tested on a single sample of chestnut shell extract spiked with gallic acid, and the result was compared with HPLC measures.

More recently, in 2020 Salamanca-Neto and coworkers proposed the use of statistical mixture design to determine the ratio of materials used in the construction of a biosensor device based on graphite oxide, platinum nanoparticles, and biomaterials, laccase and botryosphaeran, an exopolysaccharide of the (1→3)(1→6)-ß-D-glucan- obtained from *Botryosphaeria rhodina* MAMB-05. Under optimized experimental parameters by factorial design, the biosensor was applied to the voltammetric determination of chlorgenic acid, showing linear response between 0.56 and 7.3 µM and LOD of 0.18 µM. The developed laccase biosensor exhibited excellent anti-interference ability and was also used for analysis of traditional and specialty coffees beverages, and samples were also analyzed by HPLC, with very good matching [90].

**Figure 9:**
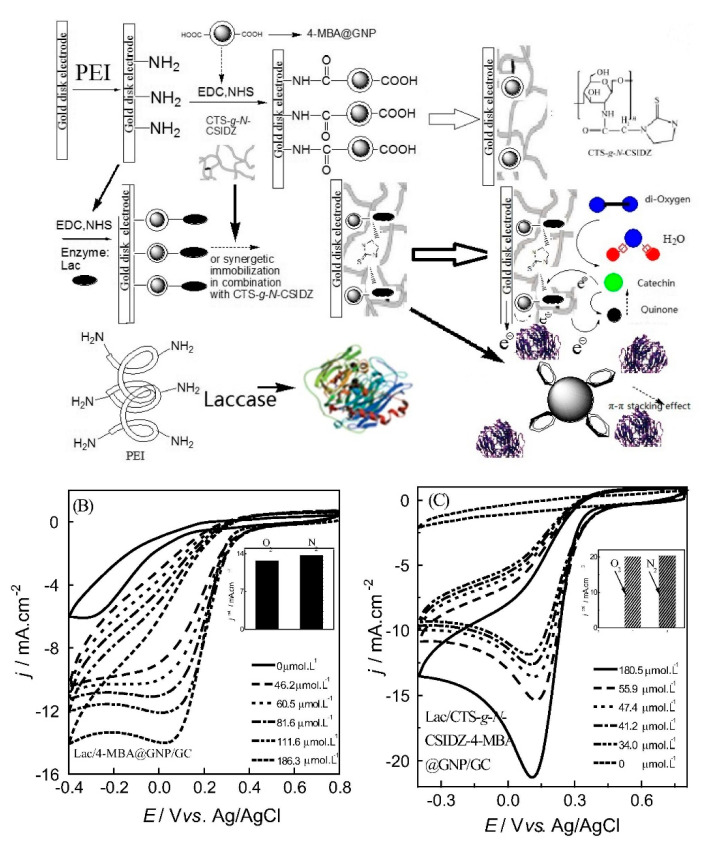
Illustrations for preparation process of Lac/CTS-g-N-CSIDZ-4-MBA@GNP/GD and its catalytic reactions on the surface of electrode. Reprinted from ref. [91] with permission of Elsevier.

In 2016, catechin was also determined using an electrode covered by nano-composite with laccase by Zeng et al. [91] (Figure 9). The nano-composite is a hybrid of chitosan-g-N-carboxymethyl-2-sulfo-4, 5-2H imidazolinone and gold nanoparticles tailored with 4-mercaptobenzoic acid, and is used as enzyme carrier to prepare glassy carbon electrode with entrapped laccase. The performance for catechin detection was evaluated by CV and chronoamperometry, in comparison with other Lac-based electrochemical sensors. The accuracy of the proposed method was demonstrated on industrial sewage samples using also HPLC. The Lac-based electrode was sensitive to catechin with high selectivity and low detection limit (16 nM). This novel sensor exhibited excellent reproducibility, long-term stability, and high tolerance to enzyme inhibitors.

##### Peroxidases

Since the early 2003, studies on peroxidase from zucchini (*Cucurbita pepo*) crude extracts, also in combination with other enzymes such as laccase, appeared for biosensing of catecholamines [92] and paracetamol [93], and were further developed in more recent years also in combination with carbon nanotubes, focusing on dopamine determination [94].

Granero and colleagues developed a peroxidase-based electrochemical sensor for resveratrol [95]. An electrode made of carbon paste mixed with ferrocene was used, and a mixture of peroxidase basic isoenzymes from *Brassica napus* was confined in the proximity of the electrode surface by means of a dialysis membrane. Hydrogen peroxide is formed by peroxidases upon reaction on resveratrol, and the amperometric sensor then follows the flux of electrons from hydrogen peroxide to ferrocene. The sensor performs well in comparison with HRP-based peroxidase systems. However, its analytical performance was very similar to that of the laccase system (Table 2).

In the early 2007, Vieira and coworkers also developed a biosensor for the detection of caffeic acid in white wine constructed by immobilization of green bean tissue homogenate—taken as a source of peroxidase—in a chitin matrix chemically crosslinked with epichlorohydrin and glutaraldehyde incorporated in a CPE. The peroxidase catalyzes the oxidation of caffeic acid to quinone in the presence of hydrogen peroxide and this product is electrochemically reduced to caffeic acid at a potential of +0.1V. A linear calibration curve was obtained for caffeic acid concentrations ranging from 20 to 200 µM, and the detection limit was found to be 2 µM. Very extensive interference studies were conducted on substances that might affect the determination of caffeic acid in white wine, including phenolic and non-phenolic compounds, and the proposed biosensor exhibited excellent selectivity toward the target molecule. Finally, the performance of the biosensor was tested in real white wine samples obtaining good recoveries, and real content results assessed by the proposed sensor agreed with those obtained by capillary electrophoresis at a 95% confidence level [96]. In a similar way, one year later the same group applied fresh bean sprouts as a source of peroxidase to develop two biosensors for the SWV determination of chlorgenic acid, by immobilizing the bean sprout homogenate in chitosan microspheres and silica, always using glutaraldehyde and epichlorohydrin for crosslinking. In this case chlorgenic acid content was determined in four coffee samples [97]. A raw material containing peroxidase was also used to setup a SWV biosensor for rosmarinic acid in ionic liquid [98]. Tissue homogenates from the pine nuts of *Araucaria angustifolia* (pinheiro do Paraná or pinheiro-Brasileiro, which contains a peroxidase in the pine kernels) were mixed with cross-linked chitosan, graphite, nujol, and an ionic liquid (BMI-Tf_2_-N), used as a support electrolyte directly within the carbon paste thus obtained. In the presence of hydrogen peroxide, the enzyme catalyzes the oxidation of rosmarinic acid to the corresponding quinone, which is then reduced back electrochemically at +0.15V vs. Ag/AgCl. The biosensor gave excellent performance, with a 73 nM LOD (Table 2), although showing a narrow linear range. Its precision was within 5%, and it gave stable signals for over 900 determinations in 300 days. Cross-reactivity was tested on caffeic acid, eriodictyol-7-O-glucoside, hesperetin, hesperidin, *m*-coumaric acid, naringenin, and naringin, and only the first two compounds were detected by the sensor. It has been validated in about 10 plant extracts from lemon balm by comparison with CE measures. The same year, the group proposed another enzymatic electrochemical biosensor for chlorgenic acid determination in coffee samples based on the ionic liquid, 1-n-butyl-3-methylimidazolium hexafluorophosphate containing dispersed iridium nanoparticles (Ir-BMI.PF_6_) and polyphenol oxidase immobilized in chitosan ionically crosslinked with oxalate. The polyphenol oxidase catalyzes the oxidation of chlorogenic acid to the corresponding quinone, which is electrochemically reduced back to this substance at +0.25V vs. Ag/AgCl [99].

Horseradish peroxidase (HRP) was used as an amperometric capsaicin biosensor by Heng et al. in 2013 [100] and 2017 [101]. The authors started in 2013 with the development of a biosensor based on a horseradish peroxidase enzyme reaction mediated by ferrocene. Both ferrocene and horseradish peroxidase are immobilized on a hydrogel membrane made of poly(2-hydroxyethyl methacrylate and capsaicin concentrations were measured at a potential of 0.22 V (vs. Ag/AgCl). The linear response range of the biosensor toward capsaicin was 2.5–99.0 μM with a LOD of 1.94 μM and the method was validated using HPLC standard method for the analysis of capsaicin. In 2017 the same authors immobilized the enzyme covalently to the surface of modified acrylic microspheres via succinimide groups preventing the leaching of the enzyme and the new biosensor gave good results in a linear response range 0.75–24.94 μM with a LOD of 0.39 μM. Also in this case, the method was validated with standard HPLC analyses.

##### Multiple Enzyme Systems

Enzymes are also key components of sensor arrays such as electronic tongues. In 2013, del Valle and coworkers developed a bioelectronic tongue for the simultaneous determination of three major phenolic compounds found in beer: ferulic, gallic, and sinapic acids. The proposed bioelectronic tongue was formed by an array of four graphite epoxy voltammetric sensors and biosensors (bare graphite-epoxy sensor and modified with tyrosinase, laccase, and copper nanoparticles), with marked cross-response toward the involved compounds, plus an artificial neural network (ANN) model able to extract meaningful data from the complex readings, overcoming signals overlapping. The proposed method was applied to determine the content of the three target phenolic acids in spiked beer samples, obtaining good recoveries [102]. One year later, Rodriguez-Mendez and coworkers developed another multisensory system formed by nanostructured voltammetric biosensors based on phenol oxidases (tyrosinase and laccase) which were incorporated into a biomimetic environment provided by a Langmuir–Blodgett film of arachidic acid. Also lutetium bisphthalocyanine (LuPc_2_) was introduced in the films to act as electron mediator, thus obtaining an array formed by three electrodes (AA/LuPc_2_, Tyr/AA/LuPc_2_, and Lac/AA/LuPc_2_). The fabricated bioelectronic tongue was able to discriminate between several phenolic compounds including one monophenol (vanillic acid), three orto-diphenols (catechol, caffeic acid, hydroquinone), and two triphenols (gallic acid and pyrogallol) through principal component analysis, with limits of detection ranging between 10 and 100 nM. The bioelectronic tongue was also applied for discrimination of musts according to their total polyphenolic index [103].

##### Enzymes Inhibited by Phenolic Compounds

**Figure 10:**
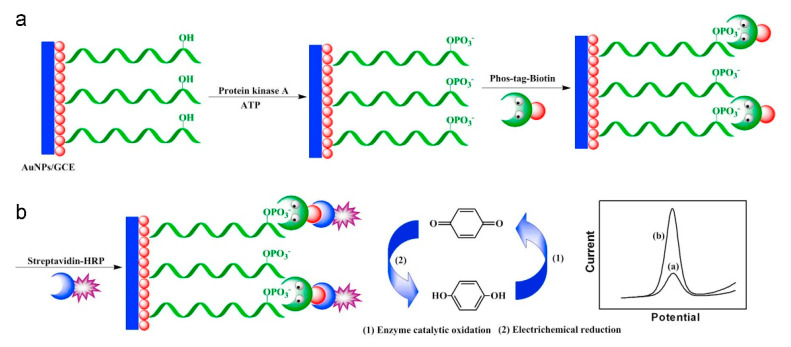
Detection of phenols as kinase inhibitors. (**a**): phosphorylation of the peptide matrix and binding of biotin; (**b**): binding of peroxidase and phenol oxidation. Reprinted from ref. [104] with permission of Elsevier.

Besides being substrates for redox enzymes, phenolic compounds may perform also as inhibitors of several enzymes, and this activity has been sporadically exploited to develop biosensors. Ellagic acid is an inhibitor of protein kinase, and a dual activity sensor has been developed, that can be used to detect it due to inhibition of kinase, or to detect kinase activity specifically inhibited by the phenol. An indirect electrochemical method was developed to obtain the sensor (Figure 10) [104]. A substrate peptide of kinase was immobilized on the surface of a glassy carbon—gold nanoparticles electrode. In the presence of kinase and ATP, the peptide is phosphorylated and can bind a phos-tag-modified biotin, which then can bind an avidin-peroxidase conjugate. Finally, the signal is obtained by the peroxidase-catalyzed oxidation of hydroquinone.

The system led to a 500 nM LOD toward ellagic acid, and a wide linear range of response (1–100 µM). Of course, the system is sensitive to any other kinase inhibitor.

#### 2.2.2. Functional Receptors of Phenolic Compounds

Phenolic compounds display a range of biological activities by interacting with specific functional receptors. This is the case of taste receptors, as several phenols such as capsaicin are typical pungent-taste compounds. The receptors can be used to develop sensors with high specificity.

**Figure 11:**
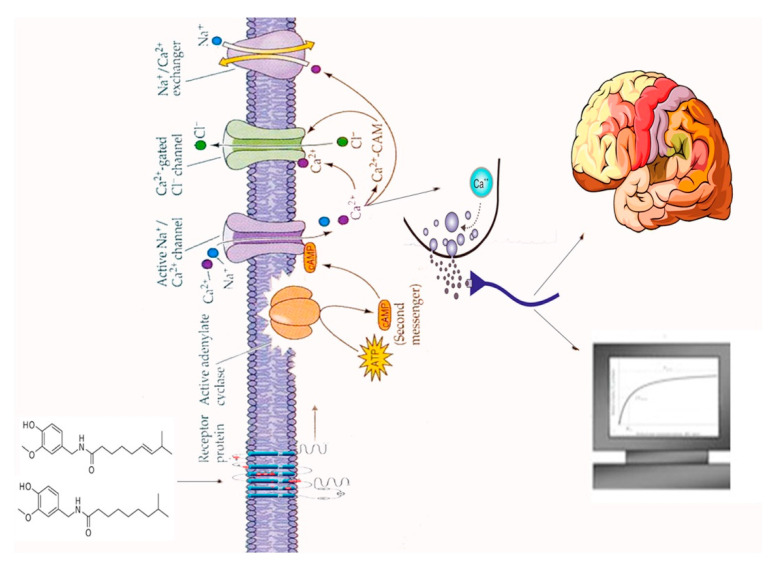
Rat tongue mucosa coupled to electrochemical system to detect pungent compounds. Reprinted from ref. [105] with permission of Elsevier.

The taste-bud tissues of SD rats (stripped rat mucosa from tongue) were used to detect pungency due to gingerol and capsaicin and to obtain a very sensitive, whole cell-based biosensor [105]. The tissues were trapped inside a microporous membrane sandwich filled with a starch-alginate gel. The sandwich was fitted over a GCE, and the current due to the release of calcium ions upon binding of the pungent compounds to the taste receptor was used for quantification, thus mimicking the taste nerve signaling process (Figure 11). The system was by far more sensitive than enzyme-based sensors, with LODs for capsaicin and gingerol of 100 fM and 1 pM respectively (Table 2). This is due to the fact that, usually, the affinities of small molecules for their membrane receptor are many orders of magnitude more favorable than those of enzymes for their substrates (K_M_s are often in the mM range, while receptors K_D_s are quite commonly pM of less). In this case, the sensitivity reflects that of rats toward capsaicin.

The αVβ3 integrin, a transmembrane protein, is known to interact with resveratrol and gingerol. It has been used as the sensing element in a biosensor for gingerol used also to measure affinities of ligands to the receptor [106]. The receptor was covalently bound to 1 µm magnetic particles activated with carboxylic groups by EDC coupling. This material was used to capture gingerol from the samples and was placed inside a disposable carbon-based electrochemical cell array, where it was kept in close proximity to the working electrode by means of 1-mm magnets surrounding the cell. DPV was then exploited to quantify the phenol. A very good detectability was achieved (260 nM, Table 2) with a two-order of magnitude linear range. The sensor was tested in ethanolic extracts of ginger rhizomes, yet not validated by comparison with a reference technique.

#### 2.2.3. Nucleic Acids

DNA and nucleic acids-related compounds have also been used sporadically as recognition elements for phenols.

DNA strands were found to strongly interact with rosmarinic acid [107]. A carbon paste electrode modified with chitosan and carbon nanotubes was covered with DNA. By this way, rosmarinic acid was determined with SWV, and a linear concentration range of 0.040–1.5 μM with a detection limit of 0.014 μM was obtained (Table 2). Rosemary extracts were analyzed, with results in agreement with those obtained with a reference HPLC method.

In a completely different approach, the protective effect of phenols on damage induced by free radicals on nucleic acids has been exploited as the signal-generating event [108]. Guanine and adenine were electro-deposed on the surface of glassy carbon electrodes, and the change of their SWV anodic peak upon radical damage was measured to quantify phenols capable to scavenge hydroxyl radicals. Gallic acid, caffeic acid, *p*-coumaric acid, and resveratrol were quantified by this way with satisfactory sensitivity in the hundred nM range. The selectivity was fully studied between the targets, and the sensor was validated on 43 samples of different flavored waters.

#### 2.2.4. Synthetic Enzymes and Receptor Mimics

Reduced mimics of enzyme catalytic sites can also be used as sensing elements. A relevant example was given by Vieira and colleagues, who used a Fe(III)Zn(II) complex that mimics the active site of red kidney bean purple acid phosphatase to detect rosmarinic acid by SWV [109].

The complex, immobilized on the electrode, catalyzes the oxidation of rosmarinic acid to its quinone, which is then determined following its back reduction by SWV. The sensitivity resembles that of enzyme-based biosensors and is micromolar, however the dynamic range is wide (Table 2) and sensor has been fully characterized. Over 900 samples from lemon balm plant extracts were tested in 300 days without any loss of signal, with a 0.16% average precision and 90–97% recoveries. All the samples were analyzed also by capillary electrophoresis with full correlation of the results (F- and t- test passed at 95% confidence interval). Moreover, the selectivity was studied over a wide set of compounds including caffeic acid, eriodictyol-7-*O*-glucoside, hesperetin, hesperidin, *m*-coumaric acid, naringenin, and naringin.

The same group synthesized a tetranuclear copper (II) complex mimicking the active site of catechol oxidase and employed it in the construction of a novel biomimetic sensor for electrochemical determination of CGA by SWV. The chlorgenic acid concentration determined through the proposed sensor was found to be linear in the range of 5.0 to 145 µM, with a detection limit of 800 nM. The proposed biomimetic sensor was subjected to extensive anti-interferences studies displaying excellent selectivity, and recovery studies and real chlorgenic acid content determination on real coffee samples were successfully performed [110].

A similar sensor was built using a cobalt (II) ethylenediamine complex to detect ellagic acid [111]. A 35 pM LOD was obtained, and the sensor resulted selective when tested toward dopamine, acetaminophen, catechol, hydroquinone, gallic acid, uric acid, ascorbic acid, and morin. It has been evaluated in spiked samples of strawberry juice, but not validated with a reference technique.

Functional receptors can also be mimicked by means of designed peptides. A peptide for non-covalent recognition of phenolic compounds was proposed in 2016 by our group. We developed a short cyclic peptide designed in silico for chlorgenic acid through a computational approach. Sensing of target phenolic compounds has been obtained through electrochemical measurements by DPV: in fact, *o*-diphenols chlorgenic acid and caffeic acid showed a significant decrease of the anodic current upon incubation with cyclic peptide, which was attributed to the binding ability of the peptide that reduces the electrochemical availability of the phenolic groups of the two acids, whereas no variation of the anodic peak was observed for monophenols *p*-coumaric acid and ferulic acid upon incubation with an excess of the cyclic peptide. Furthermore, the peptide was able to discriminate between chlorgenic acid and the other tested phenols through quenching of the intrinsic fluorescence of peptide CWWEVITFFKEC [112].

#### 2.2.5. Imprinted Polymeric Materials

Despite their usually synthetic origin, molecularly imprinted polymers (MIPs) are regarded as biomimetic materials, and MIPs-based sensors are usually included in the field of biosensors. The original idea of obtaining a binding site by a template-driven polymerization process originates from the attempt to make artificial antibodies, and the recognition process in MIPs involves the same non-covalent interactions occurring between proteins and small molecules. The field of molecular imprinting has nowadays involuted toward nanostructured materials leading to imprinted nanogels; however, also natural biopolymers can be used for the generation of biocompatible imprinted materials. For instance, zein, a corn protein, has been imprinted with curcumin to obtain imprinted magnetic nanoparticles [113]. The material was simply fabricated by adding zein and curcumin to a suspension of Fe_3_O_4_ nanoparticles in ethanol. The addition of water led to self-polymerization. Finally, the sensing material was dried over the surface of a glassy carbon electrode. The amount of curcumin in potato chips, measured with the sensor, was very well comparable with that obtained by an HPLC reference method.

Besides this first example, a very important source of imprinted polymers is represented by polysiloxanes. In 2011 Kubota and coworkers developed a novel sensitive molecularly imprinted electrochemical sensor for the selective detection of chlorgenic acid by deposition of a molecularly imprinted siloxane (MIS) onto a gold bare electrode surface previously modified with 3-mercaptopropyl siloxane. The peak current response of the MIS/Au sensor for chlorgenic acid was linear from 500 nM to 14 µM, with a detection limit of 148 nM, and the proposed method was applied for the determination of chlorgenic acid in real samples of coffee and tea, obtaining good recovery values [114]. A few years later, the same group developed an analogue MIS electrochemical sensor prepared by sol-gel process, using the acid catalyzed hydrolysis and condensation of tetraethyl orthosilicate (TEOS), phenyl triethoxysilane (PTEOS), and 3-aminopropyl-trimethoxysilane (APTMS) using caffeic acid as target molecule which was successfully tested on real matrices of red and white wines giving excellent recovery results and real content estimated values in agreement with those obtained by standard HPLC [115]. In 2016 Lima and coworkers reported a chlorgenic acid sensor based on a GCE modified with a functional platform by grafting vinyltrimethoxysilane (VTMS) in MWCNTs which were covered by a MIS film. The sensor exhibited a linear response covering a concentration ranging from 80 nM to 500 µM with a LOD of 32 nM, and the proposed method was also applied to chlorgenic acid determination in coffee, tomato, and apple samples, showing a promising potential application in food samples [116].

**Figure 12:**
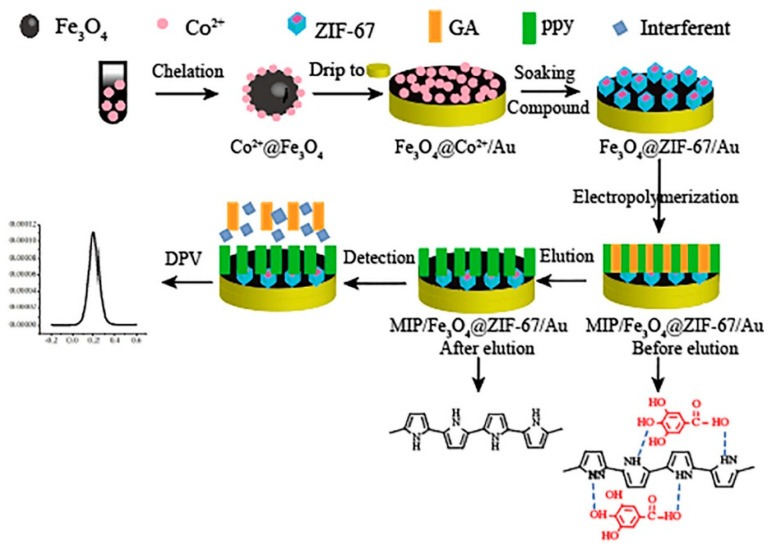
Fabrication of MIPpy/Fe_3_O_4_ @ ZIF-67/Au for electrochemical sensing of gallic acid by DPV. Reprinted from ref. [118] with permission of Elsevier.

Changing the type of molecularly imprinted material to conductive polymers, polypyrrole has been recognized as one of the most promising and frequently used conducting polymers for the synthesis of MIPs. Koirala and coworkers fabricated a potentiometric sensor by modifying pencil graphite electrodes with molecularly imprinted polypyrrole (MIPpy) synthesized by electropolymerization of pyrrole monomers at constant potential in the presence of chlorgenic acid as template molecule. The developed MIPpy sensor responded rapidly to the presence of the target molecule, exhibited an exceptionally wide linear range between 1 µM and 10 mM and the LOD was found to be 1 µM. The analytical performance of the MIPpy sensor was evaluated by employing it for the measurement of chlorgenic acid in four roasted coffee samples, and results were compared with those obtained from HPLC taken as the standard method [117]. More recently, in 2020 Huang and coworkers fabricated another MIPpy-based electrochemical sensor supported by metal-organic frameworks, taking nano-structured Co^2+^@Fe_3_O_4_ as the support point of receptor on a gold electrode, for highly sensitive detection of gallic acid. The developed sensor exhibited an excellent LOD of 0.297 pM and a wide linear range between 6 and 600 pM, also finding application in real samples analysis of black tea and green tea [118] (Figure 12).

**Figure 13:**
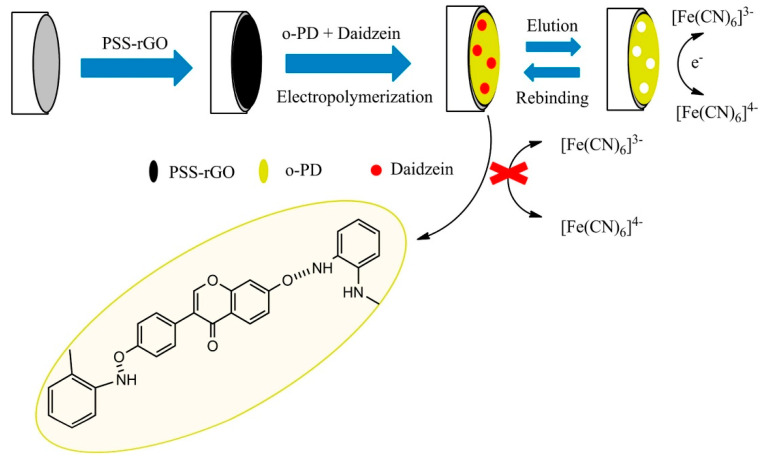
Schematic illustration of the synthesis procedure of molecularly imprinted polymer and the modified electrode construction process. Reprinted from ref. [119] with permission of Elsevier.

In 2017 Li et al. developed an electrochemical sensor with high sensitivity and selectivity for detection of daidzein using a molecularly imprinted polymer (MIP)-modified electrode [119]. The sensitive layer was prepared by electropolymerization of *o*-phenylenediamine on the surface of poly (sodium 4-styrenesulfonate)-reduced graphene oxide (PSS-rGO) modified glassy carbon electrode (PSS-rGO/GCE) in the presence of daidzein as the template molecule (Figure 13). The sensor worked in the linear range of 1.0–20.0 nM and the LOD was calculated to be 0.5 nM. Interestingly, it showed excellent selectivity with respect to other flavonoids such as puerarin, quercetin, genistein, and chrysin since the current response of MIP/PSS-rGO/GCE toward daidzein was about 6.0, 3.1, 6.9, and 5.2 times higher respectively.

The results for daidzein detection were compared with those obtained for other previously reported electrochemical sensors, thus demonstrating that the new sensor exhibits a lower LOD. Application on real samples on *pueraria* extracts and on serum samples was also verified as well as comparison with the HPLC method indicating the accuracy of the proposed method.

In 2017 Zeng et al. synthetized a novel molecularly imprinted electrochemical sensor for quercetin which was fabricated via electropolymerization of *p*-aminobenzoic acid (*p*-ABA) on a three-dimensional (3D) Pd nanoparticles-porous graphene-carbon nanotubes composite (Pd/pG-CNTs) modified glassy carbon electrode [120]. Its application is in the linear range of 0.01–0.50 µM, with a LOD of 5.0 nM. Its selectivity was demonstrated toward similar compounds such as morin, catechin hydrate, rutin, luteolin, and kaempferide. The results showed that 10-fold M and catechin, 5-fold rutin and luteolin, and 1-fold kaempferide had no interference (signal change < 5%) on the detection of 5.0 µM quercetin. A comparison of the performance of MIP/Pd/pG-CNTs/GCE with other reported quercetin sensors was also made. Application on real samples of Pule’an tablets, honeysuckle juice and red wine was also seen but no comparison with standard methods was checked.

**Figure 14:**
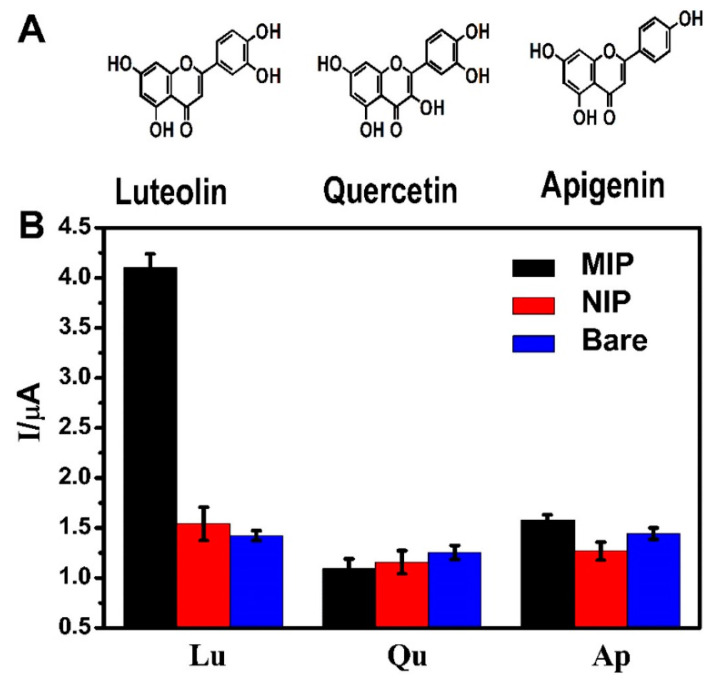
(**A**) The chemical structural formulas of luteolin and two similar structural flavonoids, from left to right: luteolin (Lu), quercetin (Qu) and apigenin (Ap). (**B**) Comparison of the DPV responses of luteolin and the structural analogs in 0.1 M PBS (pH 6.0) at MIP (black), NIP (red), and bare (blue) electrode, respectively. Luteolin concentration: 5.0 × 10^−6^ M, quercetin and apigenin concentration: 5.0 × 10^−5^ M. Incubation time: 3 min. Reprinted from ref. [121] with permission of Elsevier.

A MIP electrochemical sensor was prepared also for the detection of luteolin by Du et al. [121]. An electrochemical polymerization strategy of β-cyclodextrin (β−CD) and luteolin on an indium-tin oxide electrode, where β-CD served as the functional monomer and luteolin as the template molecule, was used for the fabrication of a MIP film-modified electrode. To verify the selectivity of the new sensor, a non-imprinted polymer (NIP)-modified electrode as the control was prepared. Luteolin could be determined in the linear response range of 0.05 μM–30 μM with a LOD of 24 nM. Performances of the new developed sensor were measured on real samples of Duyiwei capsule with recoveries of 96.0% to 105.2% and very good agreement with HPLC standard analyses was observed. Regarding the selectivity, DPV responses of luteolin and the structurally related quercetin and apigenin were considered using the MIP, NIP, and bare electrodes in 0.1 M PBS (pH 6.0) and with the following concentrations: luteolin 5 μM, quercetin and apigenin 50 μM (Figure 14).

Nasirizadeh and coworkers designed and fabricated a novel electrochemical nanosensor for determination of gallic acid based on a MIP synthesized through precipitation polymerization technique, using methacrylic acid as a functional monomer, ethylene glycol dimethacrylate as cross-linker, and gallic acid as template, and then applied the MIP in a MWCNTs-modified CPE. The proposed sensor showed a linear response range between 0.12 and 380.0 μM and LOD of 47.0 nM and was applied to determine gallic acid in apple, pineapple, orange juices, and commercial green tea drink as real samples, with satisfactory results and recoveries ranging between 98.1 and 103.3% [122].

**Table 2:** Performance of electrochemical systems with recognition elements.

Sensing System	Target	LOD	Linear Range	Non Interfering Related Compounds (Interfering Related Compounds)	Recovery	Reference Method	Real Samples (Sample Preparation)	Ref.
tyrosinase/CPE (Amp)	a selectivity study	n.d.	n.d.	resveratrol, genistein, and quercetin compared with synthetic estrogens, bisphenol A, nonylphenol, and diethylstilbestrol.	n.d.	ASTM method 9065	no	[86]
Guanine or adenine deposed on the GCE as probes of phenolic anti-oxidant activity	ascorbic acid, gallic acid, caffeic acid, coumaric acid, resveratrol	1.65 μM, 0.53 μM, 0.33 μM, 0.49 μM, 0.31 μM	2.8–14.2 μM for arachidic acid; 0.44–2.2 μM for resveratrol	fully studied between the targets	always within +/−6%	no	43 samples of different flavored water (dil. PBS)	[108]
Lac-based sensor (CV)	catechin	16 nM	8.7 μM–146.0 μM	phenols and polyphenolic compounds	n.d.	HPLC	real sample from industrial sewage (not reported)	[91]
green bean tissue homogenate (source of peroxidase) immobilized on chemically crosslinked chitin CPE (SWV)	caffeic acid	2.0 μM	20 μM–200 μM	ferulic acid, vanillic acid, syringic acid, gallic acid, *p*-coumaric acid, phenol, guaiacol, benzoic acid, (only catechin and hydroquinone produced a response)	91.0–103.1%	CE	white wine (dil.)	[96]
MIS (TEOS-PTEOS-3 APTMS) Au electrode (DPV)	caffeic acid	0.15 μM	0.500–60.0 μM	cinnamic acid, ferulic acid, *p*-coumaric acid, vanillic acid, gallic acid, 1-hydroxy-2-naphthoic acid	97.4–102.3%,	HPLC	red and white wines (dil.)	[115]
laccase on polydopamine/GCE or graphite electrode (SWV)	caffeic acid, rosmarinic acid, gallic acid	0.14 μM (caffeic acid), 0.09 μM (rosmarinic acid), 0.29 μM (gallic acid)	1–50 μM (caffeic acid), 1–20 μM (rosmarinic acid), 1–150 μM (gallic acid)	n.d.	n.d.	HPLC	chestnut shell extract, one sample compared with HPLC quite far (not reported)	[89]
CSPE/Tyr/gallic acid (Amp, CV)	Catechins	0.03 μM,	0.05-80 mM	epicatechin, epicatechin-3-gallate, gallocatechin, epigallocatechin, gallocatechingallate, epigallocatechin-3-gallate	90–96%	HPLC	black and green teas (water inf.)	[87]
graphite oxide, PtNPs, BOT and laccase (SWV)	chlorgenic acid	LOD: 0.18 μM	0.56–7.3 μM	ferulic acid, *p*-coumaric acid, caffeic acid	n.d.	HPLC	coffee (Water inf.)	[90]
bean sprout homogenate immobilized in chitosan microspheres (I) and silica (II) (SWV)	chlorgenic acid	0.8 μM (biosensor I) and 0.85 μM (biosensor II)	4.89 μM–0.32 mM (I) and 4.89 μM–48.5 μM (II)	The biosensors are sensitive to: chlorgenic acid (100%), catechol (90.5%), hydroquinone (75.0%), adrenaline (72.0%), rosmarinic acid (55.0%), caffeic acid (32.0%), adrenaline (30.0%) and l-dopa (25.0%). Esculetin, epigallocatechin gallate, ferulic acid, gallic acid, guaiacol, luteolin, *p*-coumaric acid, syringic acid, tannic acid, vanillic acid did not produce any response	96.5–102.6% (I) and 91.3–115.5% (II)	CE	4 coffee samples (water inf.)	[97]
Ir-BMI.PF_6_ and polyphenol oxidase—CS CPE (SWV)	chlorgenic acid	0.915 μM	3.48–49.5 μM	(caffeic acid causes a weak interference)	93.2–105.7%.	CE	coffee (water dispersion)	[99]
tetranuclear copper (II) complex which mimics the active site of catechol oxidase (SWV)	chlorgenic acid	0.8 μM	5.0 μM–0.145 mM	The sensor was sensitive to rosmarinic acid (100%), catechol (92.1%), chlorgenic acid (80.5%), hydroquinone (78.0%), adrenaline (71.0%), l-dopa (22.5%) and caffeic acid (12.0%). Carbidopa, epigallocatechin gallate, ferulic acid, gallic acid, guaiacol, luteolin, *p*-coumaric acid, syringic acid, tannic acid, vanillic acid did not produce any response	93.2–106.1%	CE	coffee (water inf.)	[110]
MIS (TEOS, PTEOS, APTMS) Au electrode (DPV)	chlorgenic acid	0.15 μM	0.5 μM–14 μM	caffeic acid, gallic acid, vanillic acid, catechol	94.3–107.9%	no	coffee, tea samples (water inf.)	[114]
MIS (TEOS, PTEOS, APTMS)/MWCNT-VTMS/GCE (DPV)	chlorgenic acid	0.032 μM	0.08 μM to 100 μM	gallic acid, caffeic acid	99.3–108.6%	no	coffee (water inf.), tomato, apple (Dil.)	[116]
MIPpy/PGE (pencil graphite electrode) (potentiometric)	chlorgenic acid	1μM	1 μM–10 mM	quinic acid, caffeic acid	nd	HPLC	coffee (water inf.+ dil.)	[117]
cyclic peptide CWWEVITFFKEC designed in silico (DPV and fluorescence)	chlorgenic acid (and caffeic acid)	n.d.	n.d.	ferulic acid, *p*-coumaric acid	n.d.	no	no	[112]
horseradish peroxidase enzyme (Amp)	capsaicin	1.94 μM	2.5–99.0 μM	catechol, phenol, guaiacol, 2.4-dimethylphenol, 3-chlorophenol, 3,4-dimethylphenol, 2-aminophenol, 4-chloro-3-methylphenol and resorcinol	98–102.0%	HPLC	chili samples (EtOH ext.)	[100]
immobilized horseradish peroxidase (Amp)	capsaicin	0.39 μM	0.75–24.94 μM	phenolic compounds (data not shown)	>95%	HPLC	chili fruit (EtOH ext.)	[101]
MIP (imprinted zein)/Fe_3_O_4_ NPs/GCE	curcumin	10 nM	100 nM–100 μM	n.d.	n.d.	HPLC on 3 samples	potato chips (EtOH ext.)	[113]
MIP/PSS-rGO/GCE	daidzein	0.5 nM	1.0–20.0 nM	puerarin, quercetin, genistein and chrysin	106.4–111.7%,	HPLC	pueraria lobata (EtOH ext.)	[119]
Protein kinase and immobilized peptide substrate–AuNPs GCE	ellagic acid	500 nM	1–100 μM	sensitive to any inhibitor of protein kinase	n.d.	no	not reported	[104]
ethylenediamine-Co complex (CV, Amp)	ellagic acid	35 pM	0.1–929 mM	dopamine, acetaminophen, catechol, hydroquinone, gallic acid.	in raspberry and strawberry juice. 10 mM found + 10 added. 101% avg.	no	no	[111]
Bioelectronic tongue	ferulic acid, gallic acid, *SA*	n.d.	n.d.	n.d.	average values of 103%, 103% and 106% for ferulic acid, gallic acid, *SA* respectively	no	spiked beer samples (no pretreatment requested)	[102]
taste-bud tissues of SD rats/GCE (stripped rat mucosa) (Amp)	gingerol	1 nM	2–30 nM	capsaicin (more sensitive)	n.d.	no	no	[105]
αVβ3 integrin (CV and DPV)	gingerol	260 nM	0.85–20 mM	resveratrol, genistein, and quercetin compared with synthetic estrogens, bisphenol A, nonylphenol, and diethylstilbestrol.	n.d.	no	ginger ethanolic extract (dil. PBS)	[106]
MIPpy/Fe_3_O_4_ @ ZIF-67/Au (DPV)	gallic acid	0.297 pM	6–600 pM	*p*-hydroxybenzoic acid, tannic acid, salicylic acid	89.5–118.4%	UV-Vis	black and green tea (dil.)	[118]
MIP (MAA, EGDMA) -MWCNT–CPE (DPV)	gallic acid	47.0 nM	0.12–380.0 μM	other compounds not similar to the target	98.1–103.3%	no	four different commercial juices (dil.)	[122]
MIP/ITO (DPV)	luteolin	24 nM	50 nM–30 μM	quercetin, apigenin	96.0–105.2%	HPLC	Duyiwei capsules (EtOH ext.)	[121]
MIP/Pd/pGN-CNTs/GCE (DPV)	quercetin	5.0 nM	0.01–0.50 μM	other compounds not similar to the target	90–104%	no	Pule’an tablets, honeysuckle juice and red wine (EtOH ext.)	[120]
tissue from the pine nuts of Araucaria angustifolia (containing peroxidase)-CS–IL CPE (SWV)	rosmarinic acid	72.5 nM	900 nM–4.5 mM	n.d.	97–109%	CE	about 10 plant extracts (not reported)	[98]
CPE modified with chitosan and CNTs covered with DNA (CV)	rosmarinic acid	0.014 μM	0.040–1.5 μM	n.d.	n.d.	HPLC	rosemary extract (water ext.)	[107]
Fe(III)Zn(II) complex which mimics the active site of red kidney bean purple acid phosphatase (SWV)	rosmarinic acid	2.3 mM	29.8–383 mM	caffeic acid, eriodictyol-7-*O*-glucoside, hesperetin, hesperidin, m-coumaric acid, naringenin, naringin	90–97%	CE	lemon balm plant extracts (not reported)	[109]
Laccase/Pt-Ag, AgCl electrode base (Amp)	resveratrol	1 mM	2–14 mM	caffeic acid, gallic acid, catechin, rutin, quercetin, malvidin	yes, bad in wine samples due to matrix effects. Solid phase extraction required before analysis	no	wine, with large interferences	[88]
peroxidase basic isoenzymes—ferrocene CPE (Amp)	resveratrol	0.83 μM	1–25 mM	n.d.	n.d.	no	no	[95]
bioelectronic tongue based on tyrosinase and laccase	vanillic acid, *catechol*, caffeic acid, hydroquinone, gallic acid, pyrogallol	10–100 nM	n.d.	n.d.	n.d.	no	discrimination of musts according to their Total Polyphenolic Index (dil.)	[103]

Dil.: dilution; Fil.: filtration; ext.: extraction; inf.: infusion.

## 3. Optical and Fluorimetric Sensors

### 3.1. Optical and Fluorimetric Systems without Recognition Elements

Colorimetric and fluorimetric sensors can be rather simply obtained when the phenolic target is fluorescent itself, or if separation or capture can be performed by a recognition element before exploiting a typical reaction of phenols such as the FC one. Such sensors can be developed without a specific recognition element (Table 3). As for sensors exploiting colorimetric assays, Siangproh and coworkers reported a ferulic acid paper-based colorimetric sensor preceded by thin layer chromatography (TLC): after separation, the section of the TLC plate containing ferulic acid was attached onto the patterned paper containing the colorimetric FC reagent and was eluted with ethanol, and the resulting color change was photographed and quantitatively converted to intensity using Adobe Photoshop, exhibiting a linear detection range between 0.1 mM and 0.72 mM, with a LOD of 36 μM. The paper-based sensor was used for detection and quantification of ferulic acid in three real cosmetic samples, and results compared well with those obtained by HPLC-UV method [123]. Another example of colorimetric assay was proposed in 2019 by Li and coworkers, who developed an interesting fast and accurate UV-visible spectrophotometric method to determine chlorgenic acid according to potassium ferricyanide- Fe (III) detection system by measuring absorbance at 790 nm. A good linear relationship was found between the absorbance at 790 nm and chlorgenic acid concentration in the range between 28 μM and 2.3 mM, and the proposed method was successfully applied to real samples of fermentation broth and fruits, obtaining values consistent with those from HPLC [124].

As to fluorescence, Huang and coworkers developed a label-free, turn-on fluorescence sensor for caffeic acid by the use of N-acetyl-L-cysteine-capped CdTe:Zn^2+^ quantum dots (CdTe:Zn^2+^ QDs), whose fluorescence was quenched by Fe^2+^, but was recovered in the presence of caffeic acid owing to its strong binding interaction with iron (II), leading to desorption of quenching ions from quantum dots (QDs) surface. The response of the proposed sensor was unaffected by the presence of several phenolic compounds structurally related to caffeic acid and the performance in real samples was evaluated on rapeseed, obtaining satisfactory recovery results [125]. Another interesting fluorescence-based sensor was developed using water-soluble carbon dots employed as high-performance fluorescent probes based on inner filter effect for selective and sensitive determination of chlorgenic acid which was capable of quenching QDs fluorescence linearly in the range between 0.15 and 60 µM. The performance of this inner-filter based sensor was also tested in a real honeysuckle sample, giving excellent recoveries ranging from 97.67% to 101.75% [126].

In 2020 Feng et al. fabricated a label-free fluorescent sensor for the determination of myricetin based on carbon quantum dots (CQDs) [127]. Strongly fluorescent CQDs were prepared via a green and straightforward microwave-assisted heating method using aspartic acid and urea as precursors; the fluorescence of the obtained CQDs could thus be quenched by myricetin via the inner filter effect. The new sensor had a linear detection range of 1 to 80 µM and a LOD of 18.4 nM. The selectivity of the CQDs sensor was studied considering the interfering substances chlorgenic acid, chrysophanol, rutin, daidzein, and ferulic acid, and it was established that their influence is negligible. Real samples of red wine and human serum were used to prove the applicability of the new sensor, obtaining recoveries ranging between 97.5 and 105%, but no comparison with standard analytical method was made.

A similar system was described also for curcumin, based on N,S-co-doped quantum dots [128]. The sensor was successfully used on human urine samples with a 40 nM LOD and satisfactory recovery. In a very similar way, and with similar results, P,N,B-co-doped quantum dots were also used to detect curcumin [129].

Further examples of optical sensors built without a sensing element involve specifically curcumin as a target, as this phenol is strongly fluorescent, and actually is many often used as a fluorescent probe to detect other compounds. An interesting example has been given by Kang and colleagues, who developed a covalent-rebinding system for curcumin [130]. 2-Aminoethyl diphenyl borate, which reacts selectively with the 1,3-diketone moiety of curcumin, was employed to capture curcumin and its fluorimetric detection led to a 163 nM LOD. The boronate-curcumin derivative was then applied as a fluorescent probe.

Fluorescent carbon dots were used also by Chen et al. in 2018 for determination of quercetin [131]. C-dots were synthesized by irradiation of aliphatic acids dispersed in viscous media, ionic liquids, and high boiling point solvents with capacitively coupled plasma (CCP) between regular plate electrodes in a low-pressure chamber and in presence of oxygen. In particular, they were synthesized via O_2_/CCP treatment of 1-ethyl-3-methylimidazolium dicyanamide solution of citric acid and they were used to separately probe fifteen metal ions and nine flavonoids. The two independent emissions at 430 nm (λ_ex,max_ = 330 nm) and 480 nm (λ_ex,max_ = 390 nm) of the C-dots were used to evaluate their responses to the analytes. Quercetin, hesperetin, and naringenin could quench nearly half of the 430 nm emission, but daidzein and 5-methoxyflavone passivated some unknown surface traps and enhanced the 430 nm photoluminescence intensity. The C-dots were so used to directly sense quercetin in a flavonoid-rich sample, such as *Citrus reticulata* cv. Chachiensis, which is a sun-dried peel used as a traditional Chinese medicine, called “Guang-Chen-Pi” in Chinese. For quantification, the 480 nm PL intensities were measured after equilibrium with quercetin standards in a phosphate buffer, pH 7.0. quercetin was determined using the standard addition method with a LOD of 0.5 µM in the range 2.4–119 µM. In the ethanol extracts of Guang-Chen-Pi 4.20 mg/g of quercetin were found, which was nine times higher than the value usually found in air-dried peel of *Citrus reticulata Blanco*. The authors attributed to the different species such difference in the results obtained.

**Figure 15:**
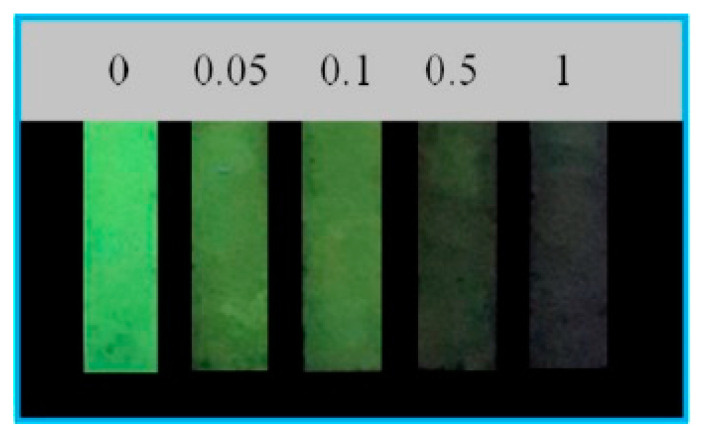
Colors of the dried test papers under the irradiation of UV light (254 nm) with different amounts of quercetin (mg mL^−1^). Reprinted with permission from ref. [132], copyright 2019, American Chemical Society.

Quercetin was also the target of the luminescent sensor developed in 2019 by Zhao et al. [132]. A novel metal organic framework {[Tb_3_(CBA)_2_(HCOO)(μ3-OH)_4_(H_2_O)]·2H_2_O·0.5DMF}n was constructed from 1,1-cyclobutanedicarboxylic acid ligand (H_2_CBA) and Tb(NO_3_)_3_·6H_2_O by solvothermal reaction at 130 °C, which displays a 3D honeycomb array with cubane cluster-based chains. The new sensor had good thermal stability and solvent/pH stability and worked in the linear range 0−993 μM with a LOD of 0.76 µM. It was used to determine the content of quercetin in onionskin and apple peel samples and the results obtained were compared with the HPLC−MS method, showing satisfactory results. Furthermore, the authors developed a portable test paper to be applied in practice. The test paper was prepared by immersing the filter paper (2.0 × 0.5 cm) in the novel metal organic framework suspension and drying it in air. After testing different concentrations of quercetin with the test paper, the luminescence changes were recorded under the irradiation of UV light (254 nm). As shown in Figure 15, the green color of the test paper became weaker and weaker and finally colorless as the concentration of quercetin increased from 0 to 1 mg mL^−1^.

In the same year, Wang et al. developed a fluorescent sensor based on carbon dots embedded metal-organic framework@molecularly imprinted polymer nanoparticles (CDs@MOF@MIP) for the optosensing of quercetin [133]. The new sensor was employed to sense trace quercetin, and its fluorescence presented a well linear decline with the increasing concentration of quercetin in the range 0–50.0 µM with a limit of detection of 2.9 nM. The authors verified the applicability of the new method to real samples of *Ginkgo biloba* extract capsules and compared the results with those obtained with the HPLC standard method. Moreover, the proposed sensor showed a certain degree of selectivity for quercetin with respect to other analogues such as isorhamnetin, epicatechin, daidzein, and rutin.

Zhu and coworkers developed a new lanthanide terbium complex, Tb (2-pyrazinecarboxylic acid)_3_(NO_3_)_3_^.^nH_2_O, synthesized by hydrothermal method, exhibiting good electrochemiluminescence behavior in the presence of triethanolamine in sodium acetate buffer solution at a GCE [134]. For sensing application, it was found that protocatuchuic acid had the ability to quench the Tb-complex electrochemiluminescence signal linearly at concentrations ranging from 1.283 pM to 385 µM, with a detection limit of 0.85 pM. The proposed method also exhibited excellent anti-interference capability in the presence of both phenolic and non-phenolic compounds.

**Figure 16:**
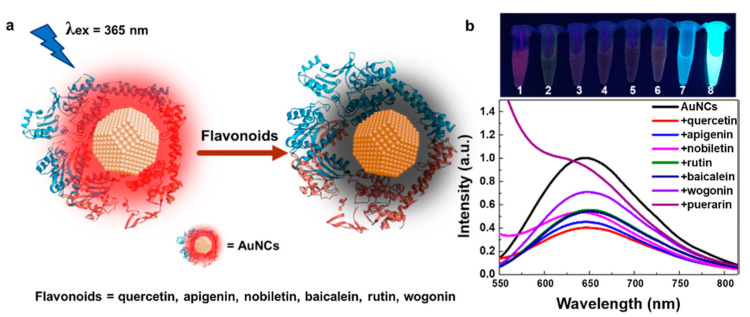
(**a**) Schematic illustration showing the AuNCs and its response to flavonoids. (**b**) Fluorescence response of AuNCs to different flavonoids. Insert: fluorescence photography of AuNCs with different flavonoids solution under UV lamp, 1: AuNCs, 2: AuNCs + quercetin (100 µg·mL^−1^), 3: AuNCs + apigenin (100 µg·mL^−1^), 4: AuNCs + nobiletin (100 µg·mL^−1^), 5: AuNCs + rutin (100 µg·mL^−1^), 6: AuNCs + baicalein (100 µg·mL^−1^), 7: AuNCs + wogonin (100 µg·mL^−1^), 8: AuNCs + puerarin (100 µg·mL^−1^). Reprinted from ref. [135] with permission of Elsevier.

An interesting approach for flavonoids detection was developed by Peng et al. in 2019 using a fluorescent gold nanocluster embedded in the structure of bovine serum albumin (BSA) [135]. The authors proposed a sensing platform based on BSA-AuNCs for the detection of various flavonoids and they determined quercetin in serum and plasma, although with potential interfering of other substances. They reached a LOD of 1.44 µg/mL for quercetin with a high recovery rate (96.7–108.6%) and they also determined the total flavonoids content in Chinese medicine Rutin tablets. The standard addition method was used in serum plasma and urine samples spiked with several different flavonoids and the results showed an excellent response and a good linearity (Figure 16).

### 3.2. Optical Sensors Exploiting Biomimetic Receptors

#### 3.2.1. Imprinted Polymers

Natural bioreceptors are rarely used in optical sensors for phenolic compounds and coupling of the sensing system to an optical or fluorimetric detection system is most often obtained by exploiting synthetic artificial receptors, as such materials can be rather simply designed embedding the signal-generating unit inside. Carbon dots, quantum dots, and molecular fluorophores can be used to deliver fluorescence or luminescence that is affected upon the interaction of the system with its phenolic target (hopefully increasing the intensity of the signal, most often decreasing it or shifting it). Several examples involve again curcumin and its fluorescence. A MIP made from acrylic acid and TEOS was grafted on MWCNTs and was used as a capture element in an HPLC-UV-Vis method for the detection of curcumin in samples of curry, ginger, and turmeric powders, and in spiked human plasma as well, with an impressive LOD of 76 pM [136]. Similarly, also methacrylic acid was used to synthesize MIPs toward curcumin [137]. Such magnetic imprinted material proved to be less sensitive than the previous sensor. By the way, the most interesting MIP developed toward curcumin has been reported by Sedghi and colleagues [138]. Differently from the other examples, the key functional monomer in this material is an acryloyl-cyclodextrin, and the monomeric unit is therefore exploiting the well-known ability of cyclodextrins to host small molecules. The affinity is then enhanced by the polymerization/imprinting process, obtained with the aid of two cross-linking co-monomers, namely TEOS and N-isopropyl-acrylamide (NIPAM), which confers to the MIP also thermoresponsive properties.

**Figure 17:**
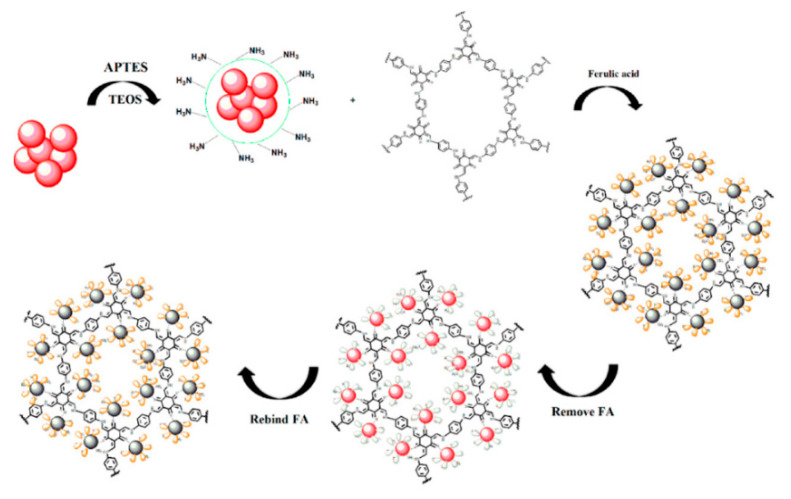
Synthesis of MIS based on quantum dot-grafted covalent organic frameworks and fluorescence behavior. Reproduced under the terms of the CC-BY Creative Commons attribution 4.0 from Reference [142], Copyright 2019.

As to fluorimetric systems involving MIPs, in 2018 Hu and coworkers proposed a fluorescence quenching sensor based on silane-functionalized carbon dots coated with caffeic acid imprinted silane-based MIPs (CDs@MIPs) [139]. Fluorescence intensity of CDs@MIPs decreased linearly with the increase of caffeic acid in the range between 0.5 and 200 μM with a LOD of 0.11 μM, and the proposed method was successfully applied to the detection of caffeic acid in spiked human plasma. One year later, a fluorescent probe based on CdTe-QDs@MIPs was proposed for selective and sensitive determination of ferulic acid, obtaining MIP shell using ferulic acid, APTES and TEOS as template, functional monomer, and crosslinker, respectively [140]. In optimal conditions, the fluorescence of CdTe-QDs@MIPs sensor exhibited extremely fast response, with a LOD of 4.4 nM, excellent linear range between 10 and 515 nM and optimal selectivity for ferulic acid compared to other structural analogues. Good recoveries were obtained in real pineapple and apple juice samples and real ferulic acid content determined through fluorescence quenching measurements coincided with those obtained by HPLC after MIP extraction, in an analogous way but changing template molecule. Shi and colleagues developed in 2019 a CdTe-QDs@MIPs fluorescent sensor for selective determination of *p*-coumaric acid exhibiting a LOD of 41 nM and linear range between 122 nM and 6.1 μM, which was applied successfully for the determination of *p*-coumaric acid in pineapple and kiwi juice samples with satisfactory recoveries [141]. In the same year, a nanomaterial of MIPs based on quantum dot-grafted covalent organic frameworks (CdSe/ZnS QD-grafted COFs) has been proposed for the determination of ferulic acid, working both as a fluorescence sensor and solid phase extraction adsorption material [142]. In the proposed nanomaterial, silane reagents had multiple functions, being used as functional monomers and crosslinkers for MIP as well as QDs surface modifiers to coordinate COFs efficiently by Schiff-base reactions, and also reacted with the phenolic hydroxyl groups of ferulic acid through non-covalent interactions (Figure 17). The fluorescence of the proposed MIP was quenched linearly at concentrations of ferulic acid ranging from 0.15 μM to 0.31 mM, exhibiting a LOD of 26 nM and both structural and functional analogues of target molecule ferulic acid could not affect the performance of the proposed sensor, which was thus tested on grain by-products samples, and results compared well with those obtained using the nanomaterial for solid phase extraction coupled to HPLC/MS.

Whereas the majority of fluorescent MIPs for phenolic compounds detection reported in literature are based on fluorescence quenching, very recently our group developed signal-on fluorescent imprinted nanoparticles for sensing of tyrosol, hydroxytyrosol, and oleuropein in aqueous olive leaves extracts [143]. Best results were obtained when using 4-vinylpiridine as functional monomer, a fluorescent co-monomer based on fluorescein, 1,4-divinylbenzene as crosslinker and tyrosol as template. The MIP showed fully satisfactory rebinding capacity of 30–35 nmol/mg and fluorescence measurements performed in water at pH 6.6 evidenced that the whole fluorescence increment occurred at extremely low concentrations of phenols, in a range between 100 fM and 100 nM, with LODs always below 1 pM for all target compounds. Finally, the sensor was tested on a real sample of olive leaves aqueous extract, and concentrations of oleuropein and related phenolic compounds determined through fluorescence measurements were in good agreement with those obtained by standard HPLC method.

#### 3.2.2. Nucleic Acids

**Figure 18:**
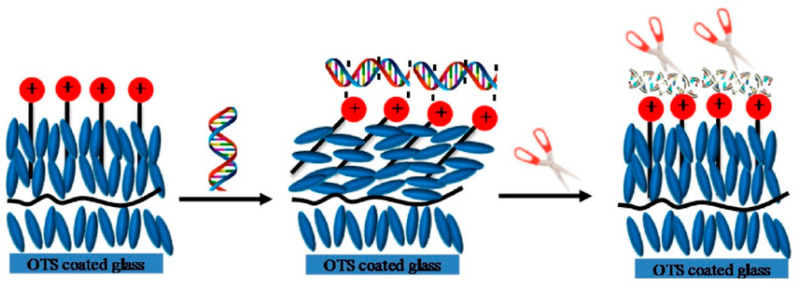
Model of the TEM_DTAB/DNA_ grid cell and the myricetin detection mechanism. Reprinted from ref. [144] with permission of Elsevier.

In 2016 Park and Munir proposed an interesting system for the determination of myricetin based on liquid crystals [144]. A 4-cyano-4′-pentylbiphenyl (a thermotropic nematic liquid crystal at room temperature) filled transmission electron microscope (TEM) grid cell was functionalized by coating with a cationic surfactant, dodecyl-trimethyl-ammonium bromide (DTAB,TEM_DTAB_), and subsequent adsorption of DNA (TEM_DTAB/DNA_) at the LC/aqueous interface. When myricetin was introduced to the TEM_DTAB/DNA_, DNA was degraded at the LC/aqueous interface and led to myricetin detection through the planar-to-homeotropic (P-H) orientational change of the 5CB observed through a polarizing optical microscope, as shown in Figure 18. Since several metal ions can affect the DNA cleavage, a study with Fe^3+^ and Cu^2+^ ions as interfering agents was conducted and the enhancement in the degradation of myricetin in the presence of Fe^3+^ and Cu^2+^ observed was comparable with the reported literatures.

### 3.3. Whole Cells Optical Sensors

**Figure 19:**
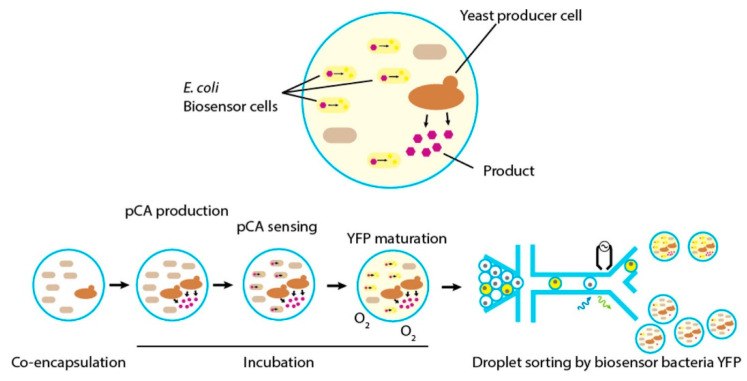
Schematic representation of the mechanism of the *E. Coli* biosensor for detection of *p*-coumaric acid producing yeast cells in picoliter droplets through YFP fluorescence detection. Reprinted with permission from ref. [146], copyright 2017, American Chemical Society.

Fluorimetric sensors have been obtained also with whole cells as the detecting and signalling units. Strauss and coworkers developed a whole cell sensor in *E. Coli* for protocatechuic acid by engineering a transcription factor regulon controlling the expression of green fluorescent protein (GFP) for induction by protocatechuic acid [145]. An autoregulated transcription factor, protocatechuic acid, was borrowed from *Acinetobacter sp* ADP1 to *E. coli* and its promoter region was adapted for activity in *E. Coli*. Confirmation of transcription factor activity was determined by linking pcaU activation to green fluorescent protein gene expression, adding exogenously supplied protocatechuic acid and screening for GFP fluorescence by flow cytometry. Going further, the authors added a second plasmid, thus providing an isopropyl-β-D-1-thiogalactopiranoside (IPTG) inducible expression of dehydroshikimate dehydratase enzyme (AsbF), which converts endogenous dehydroshikimate to protocatechuic acid. In this way the study created a microbial biosensor against both exogenously supplied and intracellularly generated protocatechuic acid. The authors found the transcription factor was responsive at protocatechuic acid concentrations in the low μM range as indicated by detectable GFP and its log-linear response region before saturation extended from 5 μM to at least 2.5 logs of concentration change. Furthermore, the developed whole-cell biosensor either showed very weak response or poor sensitivity toward other closely related benzyl family molecules like 2-hydroxy benzoate (salicylate), 4-hydroxy benzoate, vanillic acid, and vanillin. Another very interesting example of whole cell biosensor was proposed by Sommer and coworkers, who developed an *E. Coli p*-coumaric acid -responsive biosensor based on the *Bacillus subtilis* transcriptional repressor PadR, which inhibits the expression of PadC, a phenolic acid decarboxylase- and replaced the *padC* gene with the gene for the yellow fluorescent protein (YFP) [146]. Then, the optimized *E. coli*-biosensing cells were encapsulated with yeast *p*-coumaric acid-producing cells in picoliter droplets that were rapidly sorted in a microfluidic device according to the amount of *p*-coumaric acid produced using the fluorescent *E. Coli* biosensor signal when target molecule was present in the droplet (Figure 19). The *E. Coli* biosensor was also tested in the presence of different structurally similar possible interfering compounds, but only *p*-coumaric acid acted as an inducer, exhibiting a linear correlation with YFP fluorescence in the range between 0.1 and 1 mM.

## 4. Gravimetric Sensors

Microbalance devices seem scarcely described as sensors for phenolic compounds. However, a very interesting application of MIPs to a sensor has been proposed in 2014 by Gültekin and coworkers who applied MIP technology to the construction of a new caffeic acid imprinted quartz crystal microbalance nanosensor, using methacrylamidoantipyrine-iron(III) [MAAP-Fe(III)] as metal-chelating monomer to prepare selective MIP on the microbalance due to metal-chelating ability of caffeic acid [147]. Quantification of caffeic acid was based on decrease in the oscillating frequency of the QCM electrode upon binding with target molecule caffeic acid. The proposed sensor exhibited an excellent detection limit of 7.8 nM and a wide linear range between 10 nM and 1 mM and was successfully tested on real samples to determine caffeic acid levels in plant materials.

**Table 3:** Performance of optical/fluorimetric sensors.

Sensing System	Target	LOD	Linear Range	Non Interfering Related Compounds (Interfering Related Compounds)	Recovery	Reference Method	Real Samples (Sample Preparation)	Ref.
QDs-Fe^2+^ (FL)	caffeic acid	63 nM	0.14–1.4 μM	sinapoyl thiocyanate; sinapic acid; *p*-coumaric acid; cinnamic acid; pyrogallic acid; syringic acid; ferulic acid, tannins, (only EDTA and especially citric acid, giving a response higher than that of caffeic acid can interfere)	90.3–99.3%	no	rapeseed samples (MeOH/water 70:30 *v/v* ext.+ MeOH ext.)	[125]
CDs @ MIS (APTES, TEOS) (FL)	caffeic acid	0.11 μM	0.5–200 μM	other compounds not similar to the target	98.4–107.6%	no	spiked human plasma (ACN removal of proteins)	[139]
potassium ferricyanide K_3_[Fe(CN)_6_] and Fe (III) (UV-Vis)	chlorgenic acid	n.d.	28 μM and 2.3 mM	n.d.	no	HPLC	fermentation broth (EtOH ext.) and fruits (fractionated ext.)	[124]
CDs (FL)	chlorgenic acid	45 nM	0.15–60 μM	it seems to be quite selective to caffeic acid, ferulic acid, *p*-coumaric acid, quercetin	97.67–101.75%	no	honeysuckle (50% MeOH ext.+ dil.)	[126]
QDs, N,S co-doped (FL)	curcumin	40 nM	0.15–18 mM	yes, tested on 10 small molecules and 12 salts	98–102% in urine samples	no	human urine samples (not reported)	[128]
QDs, P,N,B co-doped (FL)	curcumin	68 nM	0.15–1.5 mM	10 small molecules, 12 ions and hydrogen peroxide.	95–10% in spike samples (tap water and mineral water)	no	no	[129]
2-aminoethyl diphenyl borate, which reacts selectively with the 1,3-diketone moiety of curcumin (FL)	curcumin	about 0.44 nM	n.d.	n.d.	no	no	no	[130]
MIP (AA, TEOS) grafted on MWCNTs (UV-Vis)	curcumin	76 pM	0.27 nM–3.26 μM	n.d.	94–107% in spiked samples	no	samples of curry, ginger and turmeric powders, spiked human plasma (MeOH/DMSO ext.)	[136]
MIP (MAA, magnetic) (UV-Vis)	curcumin	3.56 μM	n.d.	on 5 curcumin-related compounds	in curry powder, ginger powder and fresh ginger; 79–88%	no	curry and ginger	[137]
MIP NPs (iron oxide, TEOS, acryloyl cyclodextrin, NIPAM) (no sensing system)	curcumin	n.d.	n.d.	n.d.	n.d.	no	no	[138]
TLC + quantification through paper containing FC reagent (colorimetric)	ferulic acid	36 μM	0.1–0.72 mM	other compounds not similar to the target	n.d.	HPLC-UV	3 cosmetic samples (no pretreatment requested)	[123]
CdTe-QDs @ MIS (APTES and TEOS) (FL)	ferulic acid	4.4 nM	10–515 nM	chlorgenic acid, 4-hydroxybenzoic acid, caffeic acid, *p*-coumaric acid, vanillic acid, protocatechuic aldehyde	91.8–110.3%	Comparison between fluorescence quenching and HPLC results after MIP extraction	pineapple juice and apple juice (fil.)	[140]
MIS (APTES, TEOS) based on QD-grafted COFs (FL and solid phase extraction material)	ferulic acid	26 nM (FL) and 15 nM (HPLC/MS)	0.15 μM–0.31 mM (FL); 0.1 μM–0.1 mM (HPLC/MS)	cinnamic acid, syringic acid, caffeic acid	88–114% (FL) and 90–97% (HPLC/MS)	Comparison between fluorescence quenching and HPLC-MS results after extraction with MIPs	highland barley bran, wheat bran, corn silk, and vinasse (60% acetone ext.)	[142]
BSA-AuNCs (FL)	flavonoids (quercetin, apigenin, nobiletin, rutin, baicalein, wogonin, and puerarin)	1.44–5.07 mg/mL	0–0.2 mg/mL	other compounds not similar to the target	70.9–139%	no	serum, plasma, urine (not reported)	[135]
CQDs (FL)	myricetin	18.4 nM	1–80 μM	chlorgenic acid, chrysophanol, rutin, daidzein, ferulic acid	97.5–105%	no	red wine, human serum (dil. ACN)	[127]
TEM _DTAB/DNA_ (polarized optical microscope)	myricetin	n.d.	n.d.	other compounds not similar to the target	n.d.	no	no	[144]
Terbium-complex Tb(pzda)_3_(NO_3_)_3 ∙_ nH_2_O (Electrochemiluminescence)	protocatechuic acid	0.085 nM	0.13 nM–0.38 mM	Gallic acid trimethyl ether, trimebutine, 2-(dimethylamino)-2-phenylbutyl 3,4,5-trimethoxybenzoate maleate, curcumin, Epinephrine bitartrate, gallic acid, 3-Hydroxy-4-methoxybenzoic acid, Homovanillic acid, ferulic acid	nd	no	no	[134]
*E. Coli* biosensor producing GFP (FL)	protocatechuic acid	n.d.	sigmoidal response curve with upper and lower limits at 2000 μM and 4 μM, respectively	whole-cell biosensor either shows very weak response or poor sensitivity toward other closely related benzyl family molecules like 2-hydroxy benzoate (salicylate), 4-hydroxy benzoate, vanillic acid and vanillin	n.d.	no	no	[145]
CdTe-QDs @ MIS (APTES, TEOS) (FL)	*p*-coumaric acid	41 nM	122 nM–6.1 μM	ferulic acid, caffeic acid, cinnamic acid, chlorgenic acid, 4-hydroxybenzoic acid, vanillic acid	92.7–106.0%,	no	pineapple juice and kiwi juice (fil.)	[141]
*E. Coli* biosensor producing YFP (FL)	*p*-coumaric acid	n.d.	0.1–1 mM	cinnamic acid, caffeic acid, phloretic acid	n.d.	no	used to discriminate yeast *p*-coumaric acid-producing cells	[146]
CCP-treated C-dots (FL)	quercetin	0.5 μM	2.4–119 μM	other compounds not similar to the target	n.d.	no	Citrus reticulata cv. Chachiensis (EtOH ext.)	[131]
(MOF)-{[Tb_3_(CBA)_2_(HCOO)(μ3-OH)_4_(H_2_O)]·2H_2_O·0.5 DMF}n (colorimetric luminescence)	quercetin	0.76 μM	0−993 μM	apigenin, isorhamnetin, hesperidin, catechin, *catechol*, resorcin, hydroquinone	n.d.	HPLC-MS	onionskin and apple peel samples (MeOH ext.)	[132]
CDs@MOF@MIP (FL)	quercetin	2.9 nM	0 μM–50.0 μM	isorhamnetin, epicatechin, daidzein, rutin	n.d.	HPLC	Ginkgo biloba extract capsules (MeOH aq. Ext.)	[133]
MIPs (4-VP, DVB, fluorescein) (FL)	tyrosol, hydroxytyrosol, and oleuropein	<1 pM	1 pM–100 nM	n.d.	n.d.	HPLC	real olive leaves extracts (water inf. + dil.)	[143]

Dil.: dilution; Fil.: filtration; ext.: extraction; inf.: infusion.

## 5. Conclusions and Future Perspectives

This roundup of recent achievements on sensing systems for phenolic compounds leads to a first evidence: the area is dominated by electrochemical sensors, and any other approach plays to date a minor role. Electrochemistry is the first choice owing to the redox activity of phenols; however, it is only partly satisfactory as to selectivity in the recognition of the target analytes. In the recent decades, electro analysis underwent deep innovation, with the high impact of novel, nanostructured materials, either organic or inorganic, and composite systems combining favorable properties of both. Such systems have been widely applied also to the analysis of phenolic compounds from plants. As reported in Section 2.1, many different target phenols have been addressed by electrochemical sensors which do not exploit any specific sensing element to recognize the targets, while those selected in Section 2.2 include in their design a biological or biomimetic receptor, most often an enzyme (in 19 references) or an imprinted polymer (in 12 examples), and more rarely a functional receptor or a whole tissue containing it (3 examples) or an inorganic, metal-based enzyme mimic (3 examples). An evaluation of the detectability (expressed as the declared limit of detection) and of the dynamic range (measured as the log of the difference between the maximum and minimum concentration of the analyte within the linear range) of the different approaches is reported in Table 4.

The evaluation is clearly rough, as the number of examples is not very large, and it is different in the groups of sensors. However, as to the detectability, it appears that no advantages are obtained when electrochemical systems are equipped with recognition elements.

**Figure 20:**
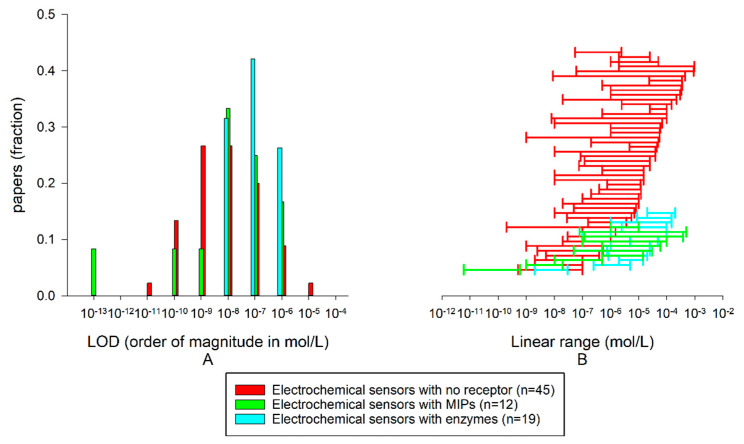
(**A**) distribution of LODs of electrochemical sensors in the papers examined in this review; (**B**) linear response ranges of electrochemical sensors in the papers examined in this review.

The sensors based on enzymes show actually a median LOD less favorable by one order of magnitude in comparison with sensors without enzymes. Moreover, as it can be appreciated from Figure 20A, no paper reports a LOD below 10 nM for enzyme sensors, while a significant fraction of unlabeled sensors can reach nano- and pico molar LODs, mostly those based on composite materials including inorganic metal components and polymeric matrices. Conversely, MIP-based systems perform better, their median LOD is close to that of unlabeled sensors, but the fraction of sensors with high sensitivity is still clearly larger in the unlabeled devices, although several examples of highly sensitive MIPs have been reported. In this case, the point could be related to losses of performance of the electrodes due to the coverage with the imprinted materials, even with the conducting ones, despite the inclusion in the design of highly active materials as CNTs and GR. As to enzymes, the limiting factor to detectability could be the affinity of the enzymes for the phenolic substrates. It is generally known that K_M_s of enzymes for their substrates are rarely sub micromolar, as the enzyme must stabilize the transition state of its reaction rather than the substrate. Under this point of view, functional receptors could be a better option, but actually only one example out of the very few found leads to detection of 1 nM gingerol [105]. Inorganic enzyme mimics seem also unable to reach low detectabilities, and in the few examples the reported LODs are micro molar.

The linear response ranges of the electrochemical sensors are visualized in Figure 20B, and their median span is reported in Table 4. Unlabeled devices show a median range width of 2.31 log units, and the linear range intervals are quite randomly distributed, without correlations with the detectabilities and also with the kind of material exploited to develop the sensor. Again, enzymes are less performing and their dynamic ranges are clearly narrower, by one order of magnitude. On the other side, imprinted materials show wider ranges, most likely owing to the heterogeneity of their binding sites; it is commonly found that MIPs contain both high- and low-affinity populations of binding sites, and this may allow to obtain a better range.

Other sensing elements as nucleic acids and peptides are found very rarely in electrochemical sensors for phenols, and the few examples do not allow evaluation of their general performance.

Coming to optical systems, and including here colorimetric and fluorimetric sensors, although less present in the literature, their detectability to phenolic compounds is comparable to that obtained with electrochemical sensors, with median LODs in the ten nanomolar range.

**Figure 21:**
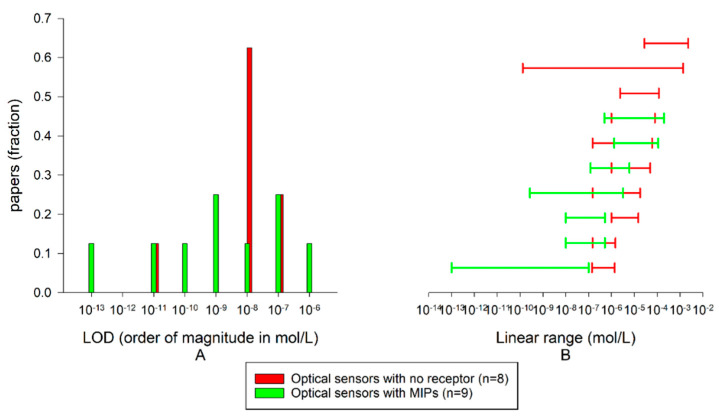
(**A**): distribution of LODs of optical sensors in the papers examined in this review; (**B**): linear response ranges of optical sensors in the papers examined in this review.

Within optical systems, a comparison can be done between sensors without specific recognition systems and MIP-based ones (See Table 4 and Figure 21).

This time the comparison is quite favorable to MIPs, as both to the number of examples with low detectabilities and to the dynamic range width. A minority of optical systems show activation of signal upon recognition of the target phenol. Several papers describe the use of bioreceptors and proteins, and their performance is not exceeding the ranges observed with the other systems.

If it is relatively easy to make a comparison between the sensors as to detectabilities and dynamic ranges, it turns out very difficult to compare the selectivity. Many systems have not been fully characterized as to selectivity, in cross-reactivity tests carried out on relevant potential interfering compounds. Thus, sensors designed without specific recognition elements are usually evaluated for selectivity toward generally occurring species that have no structural relations to the target phenols, as inorganic salts, sugars, ascorbic acid, and so on. This tendency seems to imply the evidence from the authors that such sensors could not be able to pass a selectivity test carried out on structurally related phenols. Conversely, sensors containing recognition elements are usually evaluated as to selectivity in deeper detail. In this review, we tried to focus mainly—but not only—on sensing systems whose selectivity has been tested on structural analogues of the target molecule; in fact, many examples of non-selective sensors are reported in literature, but it is often difficult to distinguish those capable of selective recognition. Another issue was that of preferentially selecting sensors and biosensors that have been tested on real samples (mainly agri-food matrices with some examples of biological matrices such as plasma and urines), especially when results obtained through the proposed methods were compared with those measured by well-established techniques that are currently being used for real analysis, such as HPLC, LC-MS, or capillary electrophoresis. This validation step with a standard technique, in fact, would be a fundamental step in the perspective of using this sensing devices in everyday analysis, thus replacing expensive techniques with fast and cheaper methodologies, also avoiding the need for highly skilled personnel to perform analysis.

Selectivity, robustness, demonstration in operational environment are closely related issues and represent the remaining challenge in the development of biosensors for phenols that could be of real impact to the end users. Considering the European Technology Readiness Levels (TLR), we can classify most of the examples reported in this review as reaching TLR level 4 (technology validate in lab) when at least some evidence of good performance in real samples and stability in time is reported. Papers lacking this evidence should be placed at level 3 (experimental proof of concept). Nevertheless, some key innovations have been achieved.

### Future Perspectives

Presently, the publication trend seems clearly in favor of chemosensors. The emerging new materials in electrochemical sensing show significant improvements toward problems as fouling and inactivation. Enzyme-based electrochemical systems, a consolidated technique, may be significantly improved if revisited and coupled with novel materials. We have reported several examples on peroxidases [92,93,94]. This field could be explored in deeper detail. Optical sensors are largely still to be developed, and molecular imprinting could still play a major role in this field to improve selectivity.

As a concluding remark toward future perspectives, the field of biosensing of phenolic compounds has been very rarely addressed by several important classes of emerging artificial receptors as aptamers and peptides, either designed or selected from biological libraries. This is rather surprising as, for instance, the contemporary literature on biosensors for pesticides is crowded by papers on aptasensors, which are absent in biosensors for natural phenols. The development of sensors based on systems different from electrochemistry and involving peptides and aptamers could represent an important field to be explored in the next years.

## Figures and Tables

**Table 4:** Comparison of target detectability of the sensors reported in this review.

	EL. Sensors without Recognition Elements	EL. Sensors with Enzymes	EL. Sensors with MIPs	Opt. Sensors without Recognition Elements	Opt. Sensors with MIPs
Median LOD	16 nM *n* = 45	500 nM *n* = 19	32 nM *n* = 12	63 nM *n* = 8	23 nM *n* = 9
Median log(C_max_-C_min_)	2.31	1.37	2.43	1.80	1.92

## Abbreviations

AdSV	adsorptive stripping voltammetry
Amp	amperometry
ACN	acetonitrile
AOC	antioxidant capacity
APTMS	(3-aminopropyl)trimethoxysilane
APTES	3-(aminopropyl) triethoxysilane
ATP	adenosine triphosphate
BSA	bovine serum albumin
CB	carbon black
CD	carbon dot
CE	capillary electrophoresis
ChAmp	chronoamperometry
COF	covalent organic framework
CPE	carbon paste electrode
CPP	capacitively-coupled plasma
CQD	carbon quantum dot
CV	cyclic voltammetry
DPV	differential pulse voltammetry
EDC	N-Ethyl-N-dimethylamminopropyl carbodiimide
EGDMA	ethylene glycol dimethacrylate
ERGO	electrochemically reduced graphene oxide
FC	Folin—Ciocalteau
FTO	fluorine doped tin oxide
*f*MIP	fluorescent molecularly imprinted polymer
GCE	glassy carbon electrode
GFP	green fluorescent protein
GO	graphene oxide
GR	graphene
HPLC	high performance liquid chroma
HRP	horseradish peroxidase
IL	ionic liquid
LOD	limit of detection
LSV	linear sweep cyclic voltammetry
MIP	molecularly imprinted polymer
MIPpy	molecularly imprinted polypyrrole
MIS	molecularly imprinted siloxane
MOF	metal organic framewrok
MWCNT	multi-walled carbon nanotube
NP	nanoparticle
PB	phosphate buffer
PBS	phosphate buffer saline
PDDA	poly (diallyldimethylammonium) chloride
PEDOT	poly(3,4-ethylene)dioxythiophene
PTEOS	phenyltriethoxysilane
QD	quantum dot
rGO	reduced graphene oxide
SPE	screen-printed electrode
SW-AdSV	square wave adsorptive stripping voltammetry
SWCNT	single-walled carbon nanotube
SWV	square wave voltammetry
TEM	transmission electron microscope
TEOS	tetraethyl orthosilicate
YFP	yellow fluorescent protein
